# Comparative transcriptomics of early petal development across four diverse species of *Aquilegia* reveal few genes consistently associated with nectar spur development

**DOI:** 10.1186/s12864-019-6002-9

**Published:** 2019-08-22

**Authors:** Evangeline S. Ballerini, Elena M. Kramer, Scott A. Hodges

**Affiliations:** 1 Department of Ecology, Evolution, and Marine Biology, University of California Santa Barbara, Santa Barbara, CA USA; 2 Current Address: Department of Biological Sciences, Sacramento State University, Sacramento, CA USA; 3 Organismic and Evolutionary Biology Department, Harvard University, Cambridge, MA USA

**Keywords:** *Aquilegia*, Petal development, RNAseq, Gene expression, Evolution, Diversification, Nectar spur

## Abstract

**Background:**

Petal nectar spurs, which facilitate pollination through animal attraction and pollen placement, represent a key innovation promoting diversification in the genus *Aquilegia* (Ranunculaceae). Identifying the genetic components that contribute to the development of these three-dimensional structures will inform our understanding of the number and types of genetic changes that are involved in the evolution of novel traits. In a prior study, gene expression between two regions of developing petals, the laminar blade and the spur cup, was compared at two developmental stages in the horticultural variety *A. coerulea* ‘Origami’. Several hundred genes were differentially expressed (DE) between the blade and spur at both developmental stages. In order to narrow in on a set of genes crucial to early spur formation, the current study uses RNA sequencing (RNAseq) to conduct comparative expression analyses of petals from five developmental stages between four *Aquilegia* species, three with morphologically variable nectar spurs, *A. sibirica*, *A. formosa*, and *A. chrysantha*, and one that lacks nectar spurs, *A. ecalcarata*.

**Results:**

Petal morphology differed increasingly between taxa across the developmental stages assessed, with petals from all four taxa being indistinguishable pre-spur formation at developmental stage 1 (DS1) and highly differentiated by developmental stage 5 (DS5). In all four taxa, genes involved in mitosis were down-regulated over the course of the assessed developmental stages, however, many genes involved in mitotic processes remained expressed at higher levels later in development in the spurred taxa. A total of 690 genes were identified that were consistently DE between the spurred taxa and *A. ecalcarata* at all five developmental stages. By comparing these genes with those identified as DE between spur and blade tissue in *A. coerulea* ‘Origami’, a set of only 35 genes was identified that shows consistent DE between petal samples containing spur tissue versus those without spur tissue.

**Conclusions:**

The results of this study suggest that expression differences in very few loci are associated with the presence and absence of spurs. In general, it appears that the spurless petals of *A. ecalcarata* cease cell divisions and enter the cell differentiation phase at an earlier developmental time point than those that produce spurs. This much more tractable list of 35 candidates genes will greatly facilitate targeted functional studies to assess the genetic control and evolution of petal spurs in *Aquilegia*.

**Electronic supplementary material:**

The online version of this article (10.1186/s12864-019-6002-9) contains supplementary material, which is available to authorized users.

## Background

**Fig. 1:**
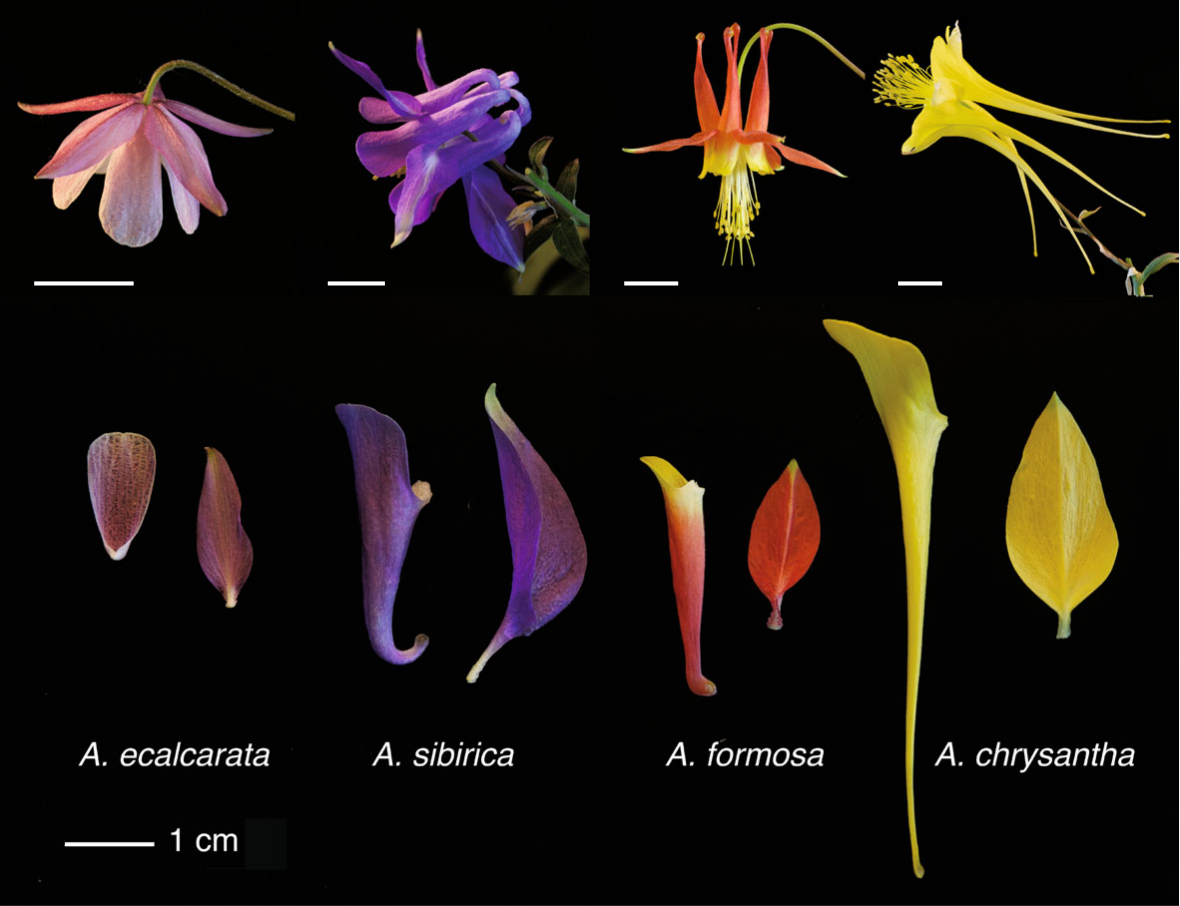
Examples of floral morphological variation in *Aquilegia* species with different pollinators. Top: entire flowers. From left to right, *A. ecalcarata*, primarily pollinated by syrphid flies; *A. sibirica*, primarily pollinated by bees; *A. formosa*, primarily pollinated by hummingbirds; *A. chrysantha*, primarily pollinated by hawk moths. Scale bars equal to 1 cm. Bottom: a dissected petal (left) and sepal (right) from each species. The adaxial surface of the *A. ecalcarata* petal is in view, whereas all other petals are more-or-less viewed in the sagittal plane with the abaxial surface in view. The adaxial surface of all sepals is in view, however the *A. sibirica* sepal is partially folded longitudinally

Approximately five to seven million years ago, nectar spurs arose in the ancestor of *Aquilegia* (Ranunculaceae), which then diversified into ∼ 70 species in two major clades: one distributed across Eurasia, and the other primarily distributed across North America [3]. Spur morphology varies substantially across the genus, ranging in length from ∼ 1-16 cm, and varying in other morphological characteristics such as the degree of curvature and color [14]. These petal characteristics contribute to pollinator specificity, which plays an important role in reproductive isolation between taxa (Fig. 1, [5]). For example, species that occur in both Eurasia and North America have the ancestral morphology of the genus — shorter, curved spurs, usually ranging in color from blue to purple — and are typically pollinated by bees. In North America, convergent evolution has lead to several lineages with red and yellow flowers, and straight, medium-length nectar spurs that are primarily pollinated by hummingbirds [27]. Multiple North American lineages that generally lack floral anthocyanins and are yellow or white have evolved long nectar spurs and are pollinated by hawk moths [27]. In addition to these three pollination syndromes, one species native to montane regions of central China, *A. ecalcarata*, has secondarily lost nectar spurs and is primarily pollinated by syrphid flies [3, 4, 25].

Given the role that nectar spurs have played in the diversification of *Aquilegia*, understanding the genetic and developmental basis of how they form is key to understanding both the initial evolution of this three-dimensional structure as well as the generation of subsequent modifications that serve as adaptations to different pollinators. Prior work has shown that development of the *Aquilegia* petal has two distinct phases [19, 29]. Phase I broadly comprises the mitotic phase of petal development in which localized cell-divisions establish the spur cup. Initially, cell divisions are dispersed throughout the petal primordium, but they cease in a wave that begins at the margins of the petal, moving basipetally and causing divisions to become concentrated in the nascent spur [19, 29]. Mitotic activity is maintained in the developing spur until the spur reaches 5-9 mm in length, but it is progressively restricted toward the spur tip, where the nectary develops [19, 29]. As cells begin to differentiate in Phase II, anisotropic cell expansion becomes a major contributor to spur shape, particularly length. Differences in spur length across *Aquilegia* species have been primarily attributed to differences in cell length, rather than cell number, although the basis of spur curvature and other aspects of shape have yet to be studied in detail [19].

In an effort to understand the genetic basis of nectar spur development in *Aquilegia*, a previous study examined gene expression differences between the petal blade and spur (previously referred to as the cup, we will simply use the terminology spur throughout this manuscript) at several early developmental stages in the horticultural variety *A. coerulea* ‘Origami’ [29]. This study ruled out a role for type I KNOX genes, which maintain cellular indeterminacy in meristematic tissue, indicating that the prolonged mitotic activity in the spur is not meristematic in nature [29]. However, a number of other genes whose homologs play a role in regulating the transition between cell proliferation and differentiation in the petals of *Arabidopsis thaliana* were highly differentially expressed between the petal blade and spur. For example, the *TEOSINTE BRANCHED/CYCLOIDEA/PCF* (*TCP*) gene, *AqTCP4*, whose homolog in *A. thaliana*, *TCP4*, controls petal size by repressing cell-division, was more highly expressed in petal blades where mitotic activity first ceases [15, 29]. In contrast, a homolog of an *A. thalianaGRF-INTERACTING FACTOR* (*GIF*) gene that controls petal size by promoting cell-division, *AqGIF1/AN3*, was up-regulated in the *Aquilegia* spur where mitotic activity is maintained [11, 29]. The most highly differentially expressed gene identified in this study is a member of the *STYLISH* (*STY*) gene family, whose homologs in *A. thaliana* are best known for their functions in carpel, rather than petal, development [8]. Subsequent analyses of this gene, *AqSTY1*, and two additional *Aquilegia* homologs in the *STY* gene family, *AqSTY2* and *Aq LATERAL ROOT PRIMORDIUM* (*AqLRP*), revealed that in addition to a conserved role in carpel development, these genes are critical to nectary development [13]. Although the *AqSTY*-like genes do not appear to function in the earliest phases of nectar spur development, these results highlight the utility of gene expression studies to identify novel candidates involved in unique roles of *Aquilegia* petal development.

The previous gene expression and functional analyses were conducted on a single *Aquilegia* cultivar, and did not address questions regarding the conservation and divergence of gene expression patterns during early petal development across the diverse morphologies in the genus *Aquilegia*. Here we conduct transcriptomic analyses of Phase I petals at five different developmental stages from four different *Aquilegia* species, *A. ecalcarata*, *A. sibirica*, *A. formosa*, and *A. chrysantha*, representing a diverse set of pollination morphologies (syrphid fly, bee, hummingbird, hawk moth) and spanning the *Aquilegia* phylogeny (including taxa from both the Eurasian and North American clades; Fig. 1). This broad sampling allowed us to take a comparative approach, combining differential expression (DE) and weighted gene correlation network analyses (WGCNA) to explore both commonalities in the genetic basis of petal development across the genus *Aquilegia* as well as taxon-specific differences. Of particular interest was identifying a core set of genes involved in spur development. To that end, a set of genes commonly DE between the three spurred species in our study, *A. sibirica*, *A. formosa*, and *A. chrysantha*, and the spurless species, *A. ecalcarata* was compared to the set of DE genes between the petal blade and spur previously conducted in *A. coerulea* ‘Origami’ [29]. This comparison revealed only 35 genes that are either more highly expressed in both *A. ecalcarata* and the petal blade or in the spurred taxa and the petal spur. In addition to these unbiased analyses, we explored the expression of *Aquilegia* homologs of genes known to regulate the transition between cell division and differentiation in *A. thaliana* petals in order determine the potential for broad functional conservation as well as any potential role in *Aquilegia* spur development.

## Results

### Developmental staging and morphology

**Fig. 2:**
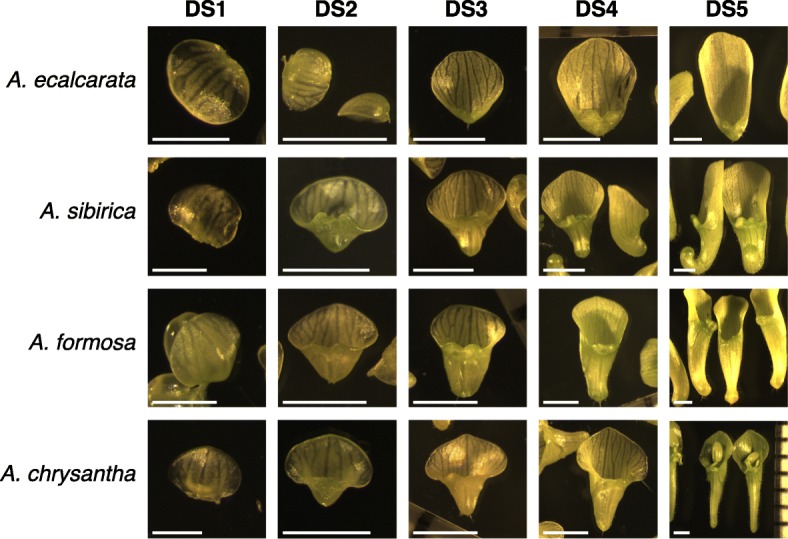
Examples of petals from each species at each developmental stage assessed. For DS1, the scale bars are 0.5 mm. For DS2-DS5, the scale bars are 1 mm. Although stamens are pictured in some photographs, all stamen material was removed prior to tissue preparation for RNA extraction

Petals were dissected from floral buds spanning Phase I of development from each of four species of *Aquilegia* - *A. ecalcarata*, *A. sibirica*, *A. formosa*, and *A. chrysantha*. Although it can be challenging to determine homologous developmental stages across taxa, a combination of petal morphology and stamen development was used to group samples across the four species into five developmental stages (DSs), DS1-DS5, with petals from at least three flowers collected for each species and stage. Representative samples of each species at each of the five developmental stages are presented in Fig. 2. The first petal stage assessed (DS1) was collected from buds approximately equivalent to floral stage 8 [1]. At this point, the morphology of petals from all four species examined was quite similar. Petals were approximately 0.5 mm wide and, although they exhibited slight concave adaxial curvature, the spur had yet to initiate. Notably, this is the first time that a pre-initiation stage petal has been sampled for RNA analysis in *Aquilegia*. The second petal stage examined (DS2) came from floral buds at approximately stage 9. At this stage, the spur had begun to form in the spurred taxa and was approximately 0.5 mm long while petals were approximately 1 mm wide in the blade region. Even at this early stage of nectar spur development, morphological differences between the taxa were already detectable, with the developing spur of *A. formosa* being wider than those of *A. sibirica* and *A. chrysantha* (Fig. 2). These differences in the spurred taxa continued through the third petal stage sampled (DS3, floral buds around early stage 10) where developing spurs continued to elongate but largely maintained their shape as established in DS2. By the fourth petal stage sampled (DS4, late stage 10 floral bud), additional morphological differences became apparent. For example, although spurs were only between 1 and 2 mm long at this stage, curvature in the *A. sibirica* petal was already being established. Some of the spurs at this stage began to appear more bulbous at the tip of the spur, indicating the incipient development of the nectary. Although *A. ecalcarata* does not develop a spur or a nectary, at DS4 a small pocket is visible below the petal attachment point. In addition to spur differences, several subtle differences in petal blade morphology across the taxa were apparent, including the shape of the blade apex and its relative length compared to the spur. The developmental gap between the fourth and fifth petal stages collected was larger than the gaps between the other stages. At the fifth time point collected (DS5, ∼floral stage 11), the spurs of the spurred taxa ranged in length from 4-7 mm and morphological differences were accentuated. *A. sibirica* spurs exhibited more extreme curvature, *A. formosa* spurs were broad through most of the length of the spur, narrowing just before the nectary, and *A. chrysantha* spurs were long and narrow. In general, this stage should represent the transition out of phase I into phase II, where cell elongation is the dominant factor affecting spur length.

### Examining patterns of petal gene expression across early developmental stages

**Fig. 3:**
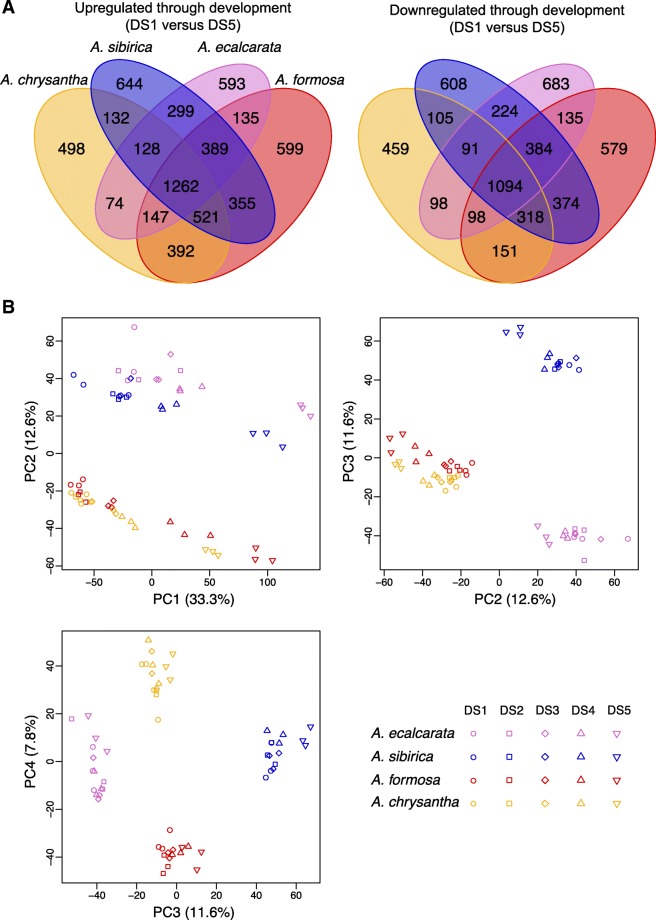
Venn diagram and PCA of genes DE in each species between DS1 and DS5. **a** Venn diagram of genes up- and down-regulated (left and right, respectively) in each species between DS1 and DS5. **b** PCA using developmentally DE genes. PCA of the genes differentially expressed in any species between DS1 and DS5 for each sample (all species and developmental stages). PC1 can be explained by developmental stage (33.3% variance explained). PC2 can be explained by phylogenetic relatedness (12.6% variance explained). PC3 captures differences between *A. ecalcarata* and *A. sibirica* (11.6% variance explained) and PC4 captures differences between *A. formosa* and *A. chrysantha* (7.8% variance explained). Key: *A. ecalcarata* = lavender, *A. sibirica* = blue, *A. formosa* = red, and *A. chrysantha* = yellow, DS1 = circle, DS2 = square, DS3 = diamond, DS4 = traingle, DS5 = inverted triangle

We started by comparing changes in the transcriptional profiles of petals between serial developmental stages (i.e., DS1 vs. DS2, DS2 vs. DS3, etc.) for each species. For *A. ecalcarata*, *A. sibirica*, and *A. chrysantha*, the differences in gene transcription were minimal between each of the first four stages, whereas there was a larger jump between DS4 and DS5 (Additional file 2, Table S2). For *A. formosa*, the most substantial expression difference occurred between DS3 and DS4, although the number of genes DE between DS4 and DS5 is also relatively high (Additional file 2, Table S2). This pattern more or less mirrors our sampling strategy in which finer sampling intervals were used between DS1 and DS4 with a larger developmental gap between DS4 and DS5. Given that there are relatively few changes across the earlier stages, for our first global analysis we focused on the end points of our sampling, bracketing Phase I of development, and identified the genes that increase or decrease in expression between DS1 and DS5. Within each species, between 5568 (*A. chrysantha*) and 6933 (*A. formosa*) genes were differentially regulated between DS1 and DS5. Counts of genes commonly up- and down-regulated between species are presented in Fig. 3a. A set of 1262 genes was commonly up-regulated across all species through development while a set of 1094 genes was commonly down-regulated across all species through development (Fig. 3a; Additional file 3). Between 498 (*A. chrysantha*) and 644 (*A. sibirica*) genes were uniquely up-regulated in just a single species across development while between 459 (*A. chrysantha*) and 683 (*A. ecalcarata*) genes were uniquely down-regulated in a single species across development (Fig. 3a). Gene Ontology (GO) enrichment analyses were conducted for the genes commonly up- and down-regulated between DS1 and DS5 in all taxa. Genes related to mitotic activity, including DNA replication, mitotic chromosome condensation, and microtubule ontologies are enriched early in development, while genes belonging to oxidation-reduction and fatty acid biosynthetic ontologies are enriched late in development (Table 1). The sets of genes up- or down-regulated in all of the spurred taxa but not *A. ecalcarata* were also identified, with 521 genes commonly up-regulated in only the spurred taxa between DS1 and DS5 and 318 genes commonly down-regulated between these stages (Additional file 4). Although the petal is generally not considered a photosynthetic organ, GO enrichment analyses on the gene sets DE in only the spurred taxa showed an enrichment of genes with GO terms related to photosynthesis were up-regulated between DS1 and DS5 (Additional file 2, Table S3).

**Table 1:** Gene Ontology (GO) categories enriched in the set of genes commonly up-regulated (Direction of expression: up) or down-regulated (Direction of expression: down) between DS1 and DS5 in all four species studied

Direction of expression	GO category	term	ontology	BH adj. *p*-value
up	GO:0055114	oxidation-reduction process	BP	2.1e-09
	GO:0016491	oxidoreductase activity	MF	1.8e-08
	GO:0016747	transferase activity, transferring acyl groups other than amino-acyl groups	MF	0.0039
	GO:0006633	fatty acid biosynthetic process	BP	0.0236
	GO:0055085	transmembrane transport	BP	0.0236
down	GO:0005840	ribosome	CC	7.6e-58
	GO:0003735	structural constituent of ribosome	MF	7.7e-57
	GO:0006412	translation	BP	3.5e-56
	GO:0005622	intracellular	CC	5.3e-32
	GO:0005634	nucleus	CC	1.7e-14
	GO:0003677	DNA binding	MF	1.7e-14
	GO:0008017	microtubule binding	MF	5.6e-11
	GO:0000786	nucleosome	CC	2.3e-10
	GO:0005871	kinesin complex	CC	4.1e-09
	GO:0006260	DNA replication	BP	6.7e-09
	GO:0003777	microtubule motor activity	MF	7.2-09
	GO:0007018	microtubule-based movement	BP	7.2e-09
	GO:0032502	developmental process	BP	2.0e-07
	GO:0004518	nuclease activity	MF	0.0010
	GO:0007076	mitotic chromosome condensation	BP	0.0019
	GO:0006351	transcription, DNA-templated	BP	0.0049
	GO:0030915	Smc5-Smc6 complex	CC	0.0054
	GO:0006355	regulation of transcription, DNA-templated	BP	0.0112
	GO:0006303	double-strand break repair via nonhomologous end joining	BP	0.0160
	GO:0015934	large ribosomal subunit	CC	0.0212

Ontology category abbreviations: BP = biological process, MF = molecular function, CC = cellular component. A GO category was deemed enriched relative to all annotated genes in the *Aquilegia* reference transcriptome if the *p*-value adjusted for multiple comparisons using the Benjamini-Hochberg (BH) method was less than 0.05

Looking across all four species, 11,258 genes showed differential expression between DS1 and DS5 in at least one species, representing a full third of predicted genes in the *Aquilegia* genome (Additional file 5). Conducting a principal component analysis (PCA) of the expression levels of these genes across all samples (all species at all developmental stages) showed that most of the variance can be explained by developmental time point (PC1, 33.3% of the variance explained; Fig. 3b). According to PC1 (as plotted versus PC2), the comparative developmental staging done across each species is fairly consistent with the expression data across taxa with a few exceptions. The *A. ecalcarata* samples assigned to DS1 have slightly higher PC1 values than the DS1 samples from the other taxa, suggesting that based on gene transcription, they may be more developmentally similar to the DS2 samples from the other taxa. Judging by PC1, the *A. chrysantha* samples assigned to DS4 and DS5 may be more transcriptionally similar to DS3 and DS4, respectively, of the other three taxa. The next several principal components are primarily explained by species differences. PC2, explaining 12.6% of the variance in this gene and sample set, primarily uncovers differences between the Eurasian (*A. ecalcarata* and *A. sibirica*) and the North American species (*A. formosa* and *A. chrysantha*; Fig. 3b). PC3 (11.6% variance explained) captures differences between *A. ecalcarata* and *A. sibirica*, while PC4 (7.8% variance explained) captures differences between *A. formosa* and *A. chrysantha*.

### Expression pattern differences between spurred taxa and spurless *A. ecalcarata*

**Fig. 4:**
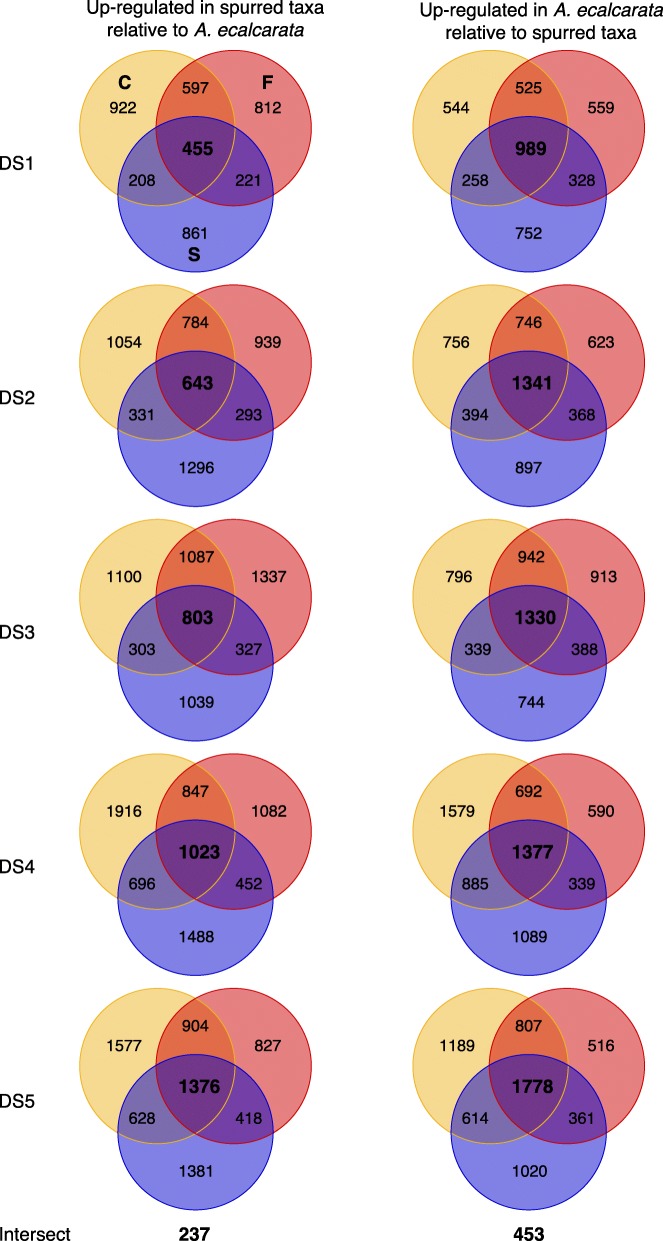
Venn diagrams of genes DE between each spurred taxon and *A. ecalcarata* at each developmental stage. Genes up-regulated in each spurred taxon relative to *A. ecalcarata* at each DS are on the left. Genes down-regulated in each spurred taxon relative to *A. ecalcarata* at each DS are on the right. Blue/S =*A. sibirica*, red/F =*A. formosa*, and yellow/C =*A. chrysantha*

In order to identify genes potentially involved in nectar spur development, differentially expressed genes between spurless *A. ecalcarata* and each spurred taxon were identified at each developmental stage. 15,588 genes are differentially expressed between *A. ecalcarata* and at least one of the spurred species during at least one developmental time point (Additional file 6). Across the first three developmental time points, a greater number of the genes identified as DE between *A. ecalcarata* and each spurred taxon are expressed at a higher level in *A. ecalcarata* than the spurred species (Table 2). By the fourth and fifth developmental stages, an approximately equal number of DE genes are expressed more highly in *A. ecalcarata* and each spurred species. DE gene sets for each of the spurred species versus *A. ecalcarata* were then compared to each other to identify genes that are commonly up- or down-regulated in the spurred taxa versus *A. ecalcarata* at each developmental stage (Fig. 4). At each developmental stage, more genes are commonly up-regulated in *A. ecalcarata* relative to all of the spurred species than up-regulated in all of the spurred species relative to *A. ecalcarata*.

**Table 2:** Number of genes up- or down-regulated in each species vs. *A. ecalcarata* at each developmental stage (DS)

	*A. sibirica*		*A. formosa*		*A. chrysantha*	
DS	up	down	up	down	up	down
1	1745	2327	2085	2401	2182	2316
2	2563	3000	2659	3078	2812	3237
3	2472	2801	3554	3573	3293	3407
4	3659	3690	3404	2998	4482	4533
5	3803	3773	3525	3462	4485	4388

The number of genes commonly up-regulated in spurred taxa gradually increases throughout development (between ∼ 25% and 41% per stage). The number of DE genes up-regulated in *A. ecalcarata* also increases across development, but the greatest increases occur between DS1 and DS2 (∼ 6%), and between DS4 and DS5 (∼ 29%). In total, 237 genes are commonly up-regulated in the spurred taxa relative to *A. ecalcarata* at all five developmental stages and 453 genes are commonly up-regulated in *A. ecalcarata* versus the spurred taxa at all five developmental stages (Fig. 4, Additional file 7). As a comparison, we also looked at the number of loci that were commonly up- or down-regulated in any one of the spurred species relative to the other three taxa (Additional file 2, Table S4). There was a common pattern in that there were more genes up-regulated in the focal species of the comparison than down-regulated (e.g., more genes are up-regulated in *A. sibirica* versus the other three taxa than down-regulated in *A. sibirica* versus the other three taxa), however, more genes were commonly DE when *A. ecalcarata* was the focal species.

**Fig. 5:**
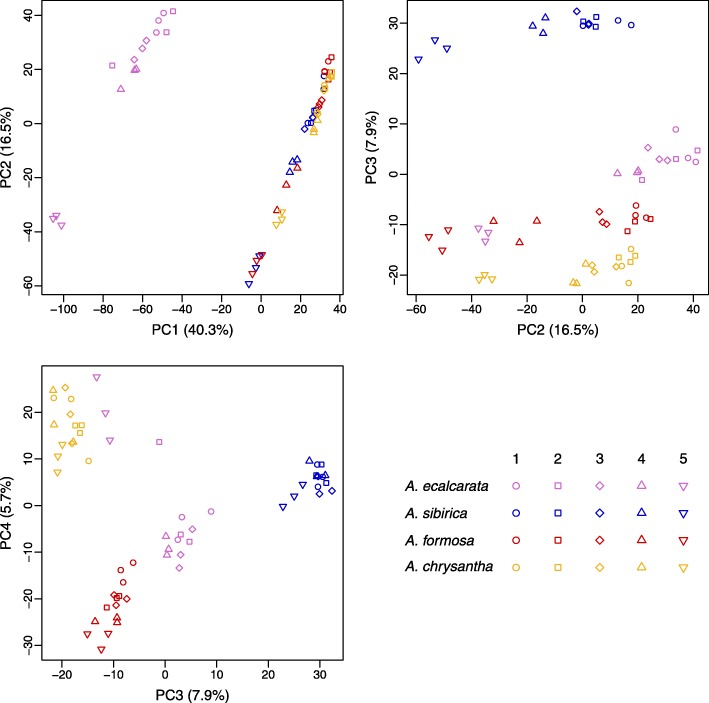
PCA of genes differentially expressed between spurred taxa and *A. ecalcarata*. PCA of the genes commonly differentially expressed between *A. ecalcarata* and each spurred species at any developmental time point for each sample (all species and developmental stages). Key: *A. ecalcarata* = lavender, *A. sibirica* = blue, *A. formosa* = red, and *A. chrysantha* = yellow, DS1 = circle, DS2 = square, DS3 = diamond, DS4 = traingle, DS5 = inverted triangle

Conducting PCA on the set of genes that were commonly DE between all spurred taxa and *A. ecalcarata* at any developmental time point (*n*=5048, Additional file 6) shows, as expected, that PC1 (40.3% variance explained) captures the difference between the spurred species and *A. ecalcarata* while PC2 (16.5% variance explained) captures common developmental differences across species (Fig. 5). PC3 (7.9% variance explained) largely captures differences between *A. sibirica* and the other taxa while PC4 (5.7% variance explained) largely captures differences between the two North American species, *A. formosa* and *A. chrysantha*. While data points for PCs 3 and 4 generally cluster by species, interestingly, the *A. ecalcarata* DS5 samples cluster with *A. formosa* in PC3 and with *A. chrysantha* in PC4, although we have no hypothesis for why this may be.

At each developmental stage, we tested for GO enrichment in the genes identified as either having higher expression in *A. ecalcarata* or commonly having higher expression in the spurred taxa versus a set of genes expressed in any species at that developmental stage (Table 3). During the earlier developmental stages (DS1-DS4), genes related to heme/iron binding and oxidoreductase activity are over-represented in the *A. ecalcarata* datasets. By DS5, many GO categories related to mitosis are enriched in the set of genes identified as significantly up-regulated in the spurred taxa.

**Table 3:** Gene Ontology (GO) categories enriched in the set of genes commonly differentially expressed between all three of the spurred taxa in this study versus *A. ecalcarata* at each developmental stage (DS)

DS	GO category	term	ontology	BH adj. *p*-value
Genes expressed more highly in *A. ecalcarata*				
1	GO:0020037	heme binding	MF	7.3-08
	GO:0005506	iron ion binding	MF	9.0e-06
	GO:0016705	oxidoreductase activity, acting on paired donors, with incorporation or reduction of molecular oxygen	MF	1.0e-05
	GO:0055114	oxidation-reduction process	BP	1.0e-05
	GO:0043531	ADP binding	MF	0.0061
	GO:0009765	photosynthesis, light harvesting	BP	0.0084
2	GO:0046983	protein dimerization activity	MF	0.0027
	GO:0020037	heme binding	MF	0.0043
	GO:0016705	oxidoreductase activity, acting on paired donors, with incorporation or reduction of molecular oxygen	MF	0.0076
	GO:0055114	oxidation-reduction process	BP	0.0085
	GO:0005506	iron ion binding	MF	0.0085
3	GO:0020037	heme binding	MF	0.0007
	GO:0016705	oxidoreductase activity, acting on paired donors, with incorporation or reduction of molecular oxygen	MF	0.0008
	GO:0055114	oxidation-reduction process	BP	0.0008
	GO:0005506	iron ion binding	MF	0.0010
4	GO:0020037	heme binding	MF	0.0100
	GO:0055114	oxidation-reduction process	BP	0.0100
5	GO:0055085	transmembrane transport	BP	3.7e-05
	GO:0042626	ATPase activity, coupled to transmembrane movement of substances	MF	0.0003
	GO:0008152	metabolic process	BP	0.0045
	GO:0006810	transport	BP	0.0046
Genes expressed more highly in spurred taxa				
2	GO:0043531	ADP binding	MF	0.0289
4	GO:0043531	ADP binding	MF	0.0041
5	GO:0005871	kinesin complex	CC	9.4e-11
	GO:0003777	microtubule motor activity	MF	9.4e-11
	GO:0007018	microtubule-based movement	BP	9.4e-11
	GO:0008017	microtubule binding	MF	9.4e-11
	GO:0006260	DNA replication	BP	3.1e-10
	GO:0003677	DNA binding	MF	1.4e-05
	GO:0000786	nucleosome	CC	2.1e-05
	GO:0007076	mitotic chromosome condensation	BP	0.0036
	GO:0009765	photosynthesis, light harvesting	BP	0.0093

GO enrichment for genes more highly expressed in *A. ecalcarata* are presented in the upper part of the table while GO enrichment of genes more highly expressed in the spurred species are presented in the lower part of the table. Ontology category abbreviations - BP : biological process, MF : molecular function, CC : cellular component. A GO category was deemed enriched relative to all annotated genes in the *Aquilegia* reference transcriptome if the *p*-value adjusted for multiple comparisons using the Benjamini-Hochberg (BH) method was less than 0.05

### Gene module analyses

**Fig. 6:**
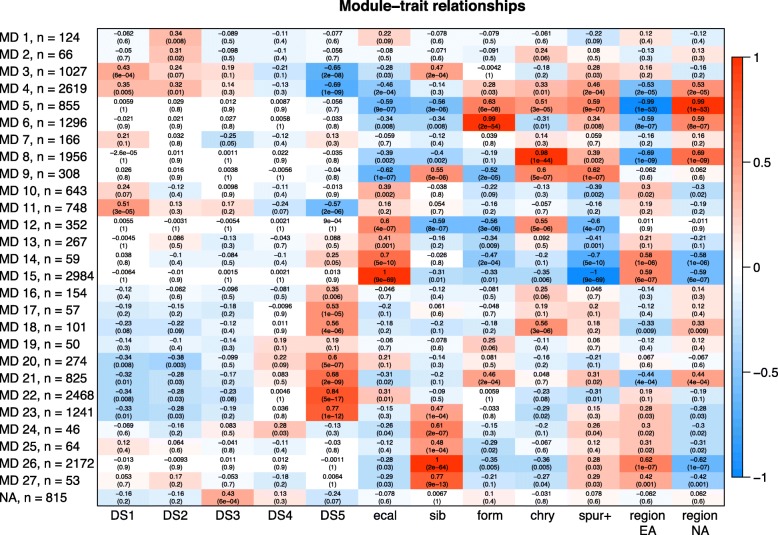
WGCNA module and trait associations. Rows correspond to the different modules formed by cluster analysis. Columns represent different traits, including developmental stage (DS1-DS5), species (ecal =*A. ecalcarata*, sib =*A. sibirica*, form =*A. formosa*, chry =*A. chrysantha*), the presence of spurs (spur+), and species region (EA = Eurasia, NA = North America). For each module-trait combination, the correlation coefficient (top value) and the *p*-value (bottom value in parentheses) of the association between the module eigengene and the trait is provided. The correlation is also color-coded, with red representing high positive correlation and blue indicating high negative correlation

**Fig. 7:**
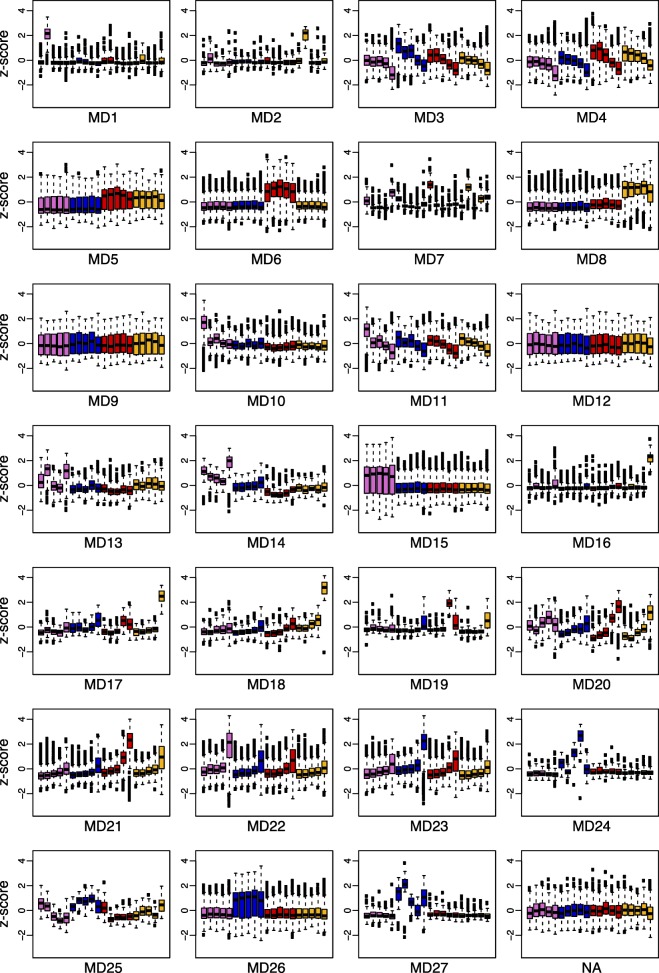
Box plots of the average z-score of the three biological replicates per species and developmental stage for genes in each WGCNA module. From left to right for each plot: *A. ecalcarata* developmental stages 1-5 (lavender), *A. sibirica* stages 1-5 (blue), *A. formosa* stages 1-5 (red), and *A. chrysantha* stages 1-5 (yellow). Z-scores for each transcript were calculated across all samples (*n*=60) before calculating the average z-score for the three biological replicates of each species and developmental stage

We used weighted gene correlation network analyses (WGCNA, [9]) to identify genetic modules (MDs) involved in petal development using the 21,790 genes expressed in any part of our data set (in any species or developmental stage). These analyses identified 27 modules ranging in size from 46 to 2984 genes (Additional file 8). 815 genes were not significantly associated with a module. Associations between modules and traits, such as developmental stage (DS1-DS5), species (*A. ecalcarata*, *A. sibirica,A. formosa*, and *A. chrysantha*), the presence or absence of spurs (*A. sibirica*, *A. formosa*, and *A. chrysantha* versus *A. ecalcarata*), and species region (Eurasian or North American, which is a proxy for phylogenetic relatedness), were identified by comparing module eigengene values with trait values (Fig. 6). In order to better visualize these module-trait relationships, boxplots of z-scores for each gene in a module are plotted as the average of the three replicates for each species and developmental time point in Fig. 7. The genes in each module were tested for GO enrichment relative to the set of expressed genes (Additional file 2, Table S5).

Generally, modules 3, 4, and 11, composed of 4394 genes, represent genes that are down-regulated through development in all taxa. Comparing the list of genes identified as significantly down-regulated in all taxa between DS1 and DS5 (*n*=1094), 16.9% are in MD3, 50.0% are in MD4, and 15.9% are in MD11 (9.0% are in MD22, and 3.0% are in MD23; Additional file 2, Table S6). While there is some overlap between the GO terms over-represented in modules 3, 4, and 11, MD3 is weighted toward genes important in ribosome assembly, MD4 has an over-representation of genes involved in DNA replication and microtubule activity, and genes involved in proteolysis and proteosome activity are overabundant in MD11 (Additional file 2, Table S5).

Modules 21-23, composed of 4534 genes, are generally marked by genes that are up-regulated across development in all taxa (Figs. 6 and 7). Again, these modules fit well with our comparative gene expression analyses. Comparing the list of genes identified as significantly up-regulated in all taxa between DS1 and DS5 (*n*=1262), 12.7% are in MD21, 49.6% are in MD22, and 21.7% are in MD23 (Additional file 2, Table S6). The genes in MD21 are expressed a bit higher in the late developmental stages of the North American species versus the Eurasian taxa and a number of gene ontologies are over-represented in this group, including oxidation-reduction, transferase activity, and fatty acid biosynthesis (Fig. 7, Additional file 2, Table S5). Composed of many genes, MD22 is marked by having slightly higher expression in *A. ecalcarata* than the spurred taxa at DS5 (Fig. 7). Genes involved in auxin response and carbohydrate metabolism are over-abundant in this module (Additional file 2, Table S5). While modules 21 and 22 have higher late-stage expression in the North American taxa and *A. ecalcarata*, respectively, MD23 has higher late stage expression in *A. sibirica* (Fig. 7). Several GO categories over-represented in this module include oxidation-reduction processes, intracellular protein transport, and iron/heme binding (Additional file 2, Table S5).

The module most associated with the presence or absence of spurs is MD15 (*R*^2^=1, p = 9e-69). This module, comprised of 2984 genes, aligns well with the differential expression analyses, as 57.0% of genes significantly up-regulated in spurred taxa at all developmental stages are in this module (11.8% are in MD4, 10.1% are in MD8, and 6.3% are in MD26) and 89.2% of genes significantly up-regulated in *A. ecalcarata* at all developmental stages relative to spurred taxa are in this module (Additional file 2, Table S7). Although this module is highly associated with the presence or absence of spurs, no gene ontologies are significantly enriched in this module. In addition to modules associated with development and the presence or absence of nectar spurs, eigengenes of several modules are highly correlated specifically with one of the spurred taxa. MD26 is highly correlated with *A. sibirica* (*R*^2^=1, p = 2e-64), MD6 is highly correlated with *A. formosa* (*R*^2^=0.99, p = 2e-54), and MD8 is highly correlated with *A. chrysantha* (*R*^2^=0.98, p = 1e-44). The set of genes uniquely DE in only one of the spurred taxa versus *A. ecalcarata* at any developmental stage was identified. 74% of the genes in MD26 are uniquely DE in *A. sibirica* (33% of all *A. sibirica* uniquely DE genes are in MD26; Additional file 2, Table S9). Of the genes in MD6, 69% are uniquely DE in *A. formosa* (22.8% of all *A. formosa* uniquely DE genes; Additional file 2, Table S8). Uniquely DE genes in *A. chrysantha* comprise 74% of the genes in MD8 (26% of all *A. chrysantha* uniquely DE genes; Additional file 2, Table S10). The genes in the *A. sibirica* and *A. formosa* specific modules (MD26 and MD6, respectively) are not enriched for any GO categories, however, the *A. chrysantha* specific module is enriched for genes involved in DNA repair, telomere maintenance, and DNA helicase activity.

### Comparison of genes DE between the spurred taxa and *A. ecalcarata* with genes DE between the developing spur and blade tissues in *A. coerulea* ‘Origami’

A prior study sought to identify genes important for nectar spur development in the horticultural variety *A. coerulea* ‘Origami’ by comparing gene expression between two regions of developing petals: the distal tip of the developing nectar spur (cup) and the petal blade [29]. *A. coerulea* ‘Origami’ has relatively long nectar spurs, similar to those of hawkmoth-pollinated species, and these comparisons between developing spur and blade were made at two developmental times points, when petals were 1 mm and 3 mm long. As this prior study was conducted using an earlier *Aquilegia* genome annotation, we re-analyzed the data using the *A. coerulea* “Golsdmith” v3.1 genome annotation (https://phytozome.jgi.doe.gov/, [2]). Counts of genes DE either developmentally or tissue-specifically are summarized in Supplemental Tables S11 and S12 (Additional file 2). Focusing on DE genes between the blade and spur cup tissue, at the 1mm stage, 490 genes were more highly expressed in the blade and 280 genes were more highly expressed in the spur cup (Additional file 2, Table S12; Additional file 9). At the 3 mm stage, 1178 genes were more highly expressed in the blade and 767 genes were more highly expressed in the spur cup (Additional file 2, Table S12; Additional file 10). The results of this reanalysis are quite similar to the original analysis [29] and exhibit a pattern common to that seen in the comparison of *A. ecalcarata* to the spurred taxa at early stages (DS1-DS3): more genes were commonly up-regulated at both the 1 mm and 3 mm stages in the spurless tissue sample (blade) than in the sample with spur tissue (spur cup; Additional file 2, Table S13).

These DE gene sets were compared to the lists of genes commonly DE between the spurred taxa and *A. ecalcarata* at roughly comparable developmental stages (DS3 ∼ 1mm, DS4 ∼ 3mm). Since *A. ecalcarata* petals consist primarily of blade tissue, we hypothesized that there would be more genes commonly up-regulated between whole *A. ecalcarata* petals and the *A. coerulea* ‘Origami’ blade tissue (collectively referred to as the ‘blade’ class), and between entire petals of the spurred taxa and the *A. coerulea* ‘Origami’ spur tissue (collectively referred to as the ‘spur’ class), compared to the opposite combinations (blade tissue/petals of spurred taxa and spur tissue/petals of *A. ecalcarata*). This is indeed the case, however, there are some genes that are more highly expressed in the blade tissue and the spurred taxa or in the spur tissue and in *A. ecalcarata* (Additional file 2, Table S13).

**Fig. 8:**
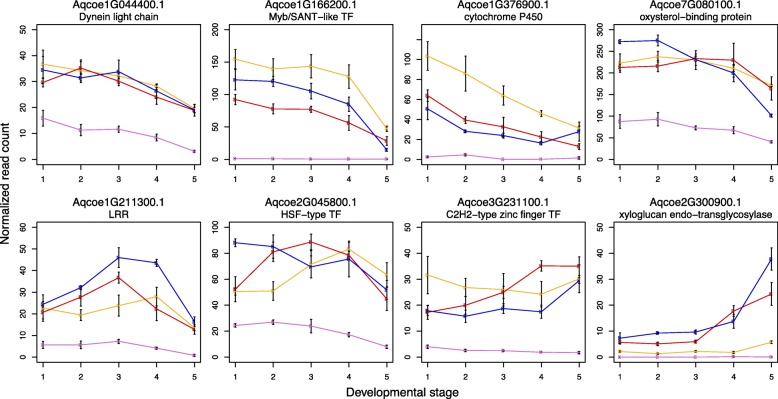
Genes up-regulated in spurred taxa and in the spur cup of *A. coerulea* “Origami”. Mean normalized read counts across three biological replicates for each species and developmental stage. The error bars represent the standard error of the three biological replicates used to calculate the mean. Key: *A. ecalcarata* = lavender, *A. sibirica* = blue, *A. formosa* = red, and *A. chrysantha* = yellow

For the purposes of identifying genes that make up a potential core module for spur development, we were particularly interested in genes that fall into the ‘blade’ and ‘spur’ classes defined above. Comparing genes that are more highly expressed in *A. ecalcarata* than the spurred taxa at all developmental stages (*n*=453) to those that are expressed more highly in the blade than the spur at both the 1mm and 3mm time point (*n*=326) reveals only 27 common genes (Tables 4 and 5, Additional file 11). Looking at the intersect of genes that are more highly expressed in all of the spurred taxa at the stages we assessed (*n*=237) with those that are expressed higher in the developing spur at both the 1 mm and 3 mm stages (*n*=190) reveals an even smaller set of only 8 common genes (Tables 4 and 5, Additional file 11). Of the 8 genes more highly expressed in ‘spur’ class, four show a general trend of decreasing through development in the spurred taxa, especially between DS4 and DS5 (Fig. 8). These genes encode a dynein light chain protein, a myb/SANT-like transcription factor, a cytochrome P450 protein, and an oxysterol-binding protein. Two genes, encoding a leucine rich repeat (LRR) protein and a heat shock factor (HSF) transcription factor, show a pattern of increasing expression in the early stages assessed followed by a decrease, while another gene encoding a C2H2-type zinc finger transcription factor has more steady expression throughout development. One gene, encoding a xyloglucan endo-transglycosylase protein, has relatively low expression in the early developmental stages with an uptick in expression moving into DS5 in the spurred taxa, especially in *A. sibirica* and *A. formosa* (Fig. 8). Interestingly, the *STY* homologs, which were among the most strongly differentially expressed loci in the blade to cup comparison and which were later found to be critical to nectary development, were not among the genes overlapping with the spurred to unspurred comparison. During later stages of petal development, however, there is some divergence between the *STY* homolog expression among the species sampled here, with *A. ecalcarata* showing lower expression than the other three (Additional file 1, Figure S2). Among the 27 genes that are more highly expressed in the ‘blade’ class, several transcription factors stand out. These include a gene in the NAC transcription factor family with similarity to ANAC034 (*AtLOV1*), an OVATE Family Protein (OVP) with similarity to *AtOFP4*, and a gene with homology to the cryptochrome 2-interacting basic helix-loop-helix (*CIB*) transcription factor *AtCIB1* (Table 5 and Additional file 1, Figure S3).

**Table 4:** The number of genes identified as differentially expressed (DE) between two tissue classes, ‘blade’ and ‘spur’, in the current study and in Yant et al., (2015)

Tissue	Current Study	Yant et al., 2015	Intersect of
Class	DS1 through DS5	1 mm and 3 mm	studies
‘blade’	*A. ecalcarata*	*A. coerulea* “Origami” blade	
	453	326	27
‘spur’	spurred taxa	*A. coerulea* “Origami” spur	
	237	190	8

In the current study, ‘blade’ tissue refers to the *A. ecalcarata* sample and ‘spur’ tissue refers to the three spurred taxa. In the Yant et al., (2015) study, the ‘blade’ tissue is blade tissue from *A. coerulea* “Origami” and the ‘spur’ tissue is spur tissue from *A. coerulea* “Origami”

**Table 5:** The list of genes identified as consistently differentially expressed between ‘spur’ class and ‘blade’ class tissue in this study and in Yant et al., (2015) and their protein family (Pfam) definitions

Expressed higher in	Locus Name	Protein family (Pfam) definition
‘spur’ class	Aqcoe1G044400	PF01221 - Dynein light chain type 1
	Aqcoe1G166200	PF13837 - Myb/SANT-like DNA-binding domain
	Aqcoe1G376900	PF00067 - Cytochrome P450
	Aqcoe1G211300	PF00560 - Leucine Rich Repeat; PF13855 - Leucine rich repeat
	Aqcoe2G045800	PF00447 - HSF-type DNA-binding
	Aqcoe2G300900	PF06955 - Xyloglucan endo-transglycosylase (XET) C-terminus
		PF00722 - Glycosyl hydrolases family 16
	Aqcoe3G231100	PF13912 - C2H2-type zinc finger
	Aqcoe7G080100	PF01237 - Oxysterol-binding protein
		PF15413 - Pleckstrin homology domain
‘blade’ class	Aqcoe1G171000	NA
	Aqcoe1G224200	PF05097 - Protein of unknown function (DUF688)
	Aqcoe2G096600	PF01699 - Sodium/calcium exchanger protein
	Aqcoe2G194000	NA
	Aqcoe2G249500	PF13202 - EF hand; PF07885 - Ion channel
	Aqcoe2G272700	PF00582 - Universal stress protein family
	Aqcoe3G124200	PF00295 - Glycosyl hydrolases family 28
	Aqcoe3G392900	PF00560 - Leucine Rich Repeat
		PF13855 - Leucine rich repeat
	Aqcoe3G424300	NA
	Aqcoe4G007000	PF00083 - Sugar (and other) transporter
	Aqcoe4G086200	PF00931 - NB-ARC domain
	Aqcoe4G229000	PF00931 - NB-ARC domain
	Aqcoe5G014800	PF00891 - O-methyltransferase
		PF08100 - Dimerisation domain
	Aqcoe5G029700	PF08263 - Leucine rich repeat N-terminal domain
		PF00069 - Protein kinase domain
		PF00560 - Leucine Rich Repeat
		PF13855 - Leucine rich repeat
	Aqcoe5G126100	PF00069 - Protein kinase domain
	Aqcoe5G143900	NA
	Aqcoe5G325200	PF09261 - Alpha mannosidase, middle domain
		PF01074 - Glycosyl hydrolases family 38 N-terminal domain
		PF07748 - Glycosyl hydrolases family 38 C-terminal domain
	Aqcoe5G408100	PF00201 - UDP-glucoronosyl and UDP-glucosyl transferase
	Aqcoe5G474100	PF04844 - Transcriptional repressor, ovate
	Aqcoe6G001700	PF02365 - No apical meristem (NAM) protein family
	Aqcoe6G025500	PF00171 - Aldehyde dehydrogenase family
	Aqcoe6G122000	PF00010 - Helix-loop-helix DNA-binding domain
	Aqcoe7G007800	PF00612 - IQ calmodulin-binding motif
		PF13178 - Protein of unknown function (DUF4005)
	Aqcoe7G015100	PF00201 - UDP-glucoronosyl and UDP-glucosyl transferase
	Aqcoe7G086200	PF04784 - Protein of unknown function, DUF547
		PF14389 - Leucine-zipper of ternary complex factor MIP1
	Aqcoe7G318900	PF07714 - Protein tyrosine kinase
	Aqcoe7G355800	PF00450 - Serine carboxypeptidase

### Expression of homologs to *Arabidopsis* genes known to be involved in early petal development

In *Arabidopsis*, many genetic factors have been identified that influence petal shape. These often function by regulating the timing of the transition from cell proliferation to cell differentiation (reviewed in [6]). Genes identified as playing a role in controlling this transition can broadly be broken down into those that promote proliferation versus those that repress proliferation. The cell proliferation activators start off broadly expressed across the primordium but they become down-regulated in a basipetal wave that is complementary to an opposing pattern of up-regulation of the cell proliferation repressors. As the early phase of nectar spur development in *Aquilegia* involves prolonging localized cell divisions in the spur, we explored the expression of the *Aquilegia* homologs to these *Arabidopsis* candidates in our dataset to see if their expression patterns are generally consistent with a conserved role in *Aquilegia* petal development or differ between *A. ecalcarata* and the spurred taxa.

**Fig. 9:**
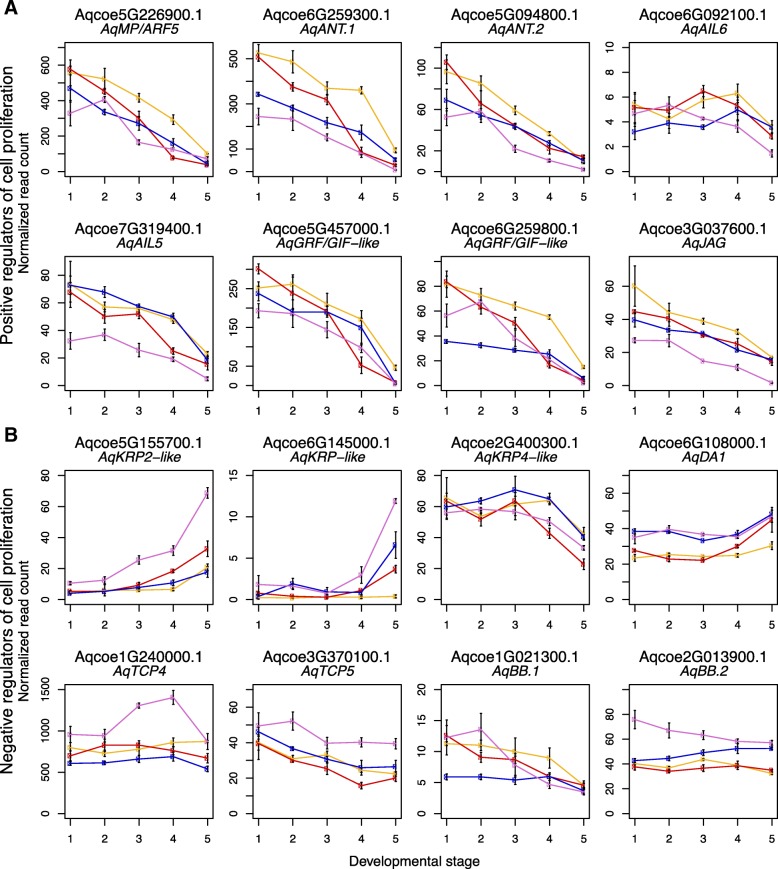
Expression of homologs of petal cell developmental regulators. **a** Normalized read counts of *Aquilegia* homologs of positive regulators of *A. thaliana* cell proliferation across development. **b** Normalized read counts of *Aquilegia* homologs of negative regulators of *A. thaliana* cell proliferation across development. For each plot, mean normalized read counts across three biological replicates for each species and developmental stage are presented. The error bars represent the standard error of the three biological replicates used to calculate the mean. Key: *A. ecalcarata* = lavender, *A. sibirica* = blue, *A. formosa* = red, and *A. chrysantha* = yellow

*Arabidopsis* genes that positively regulate cell proliferation include *AUXIN-REGULATED GENE INVOLVED IN ORGAN GROWTH* (*ARGOS*), *MONO-PTEROS/AUXIN RESPONSE FACTOR 5* (*MP/ARF5*), *AINTEGUMENTA* (*ANT*), *AINTEGUMENTA-LIKE 6* (*AIL6*), various members of the *GROWTH-REGULATING FACTOR* (*GRF*) family, and *JAGGED* (*JAG*) (reviewed in [6]). Homologs to all of these genes (see Additional file 1, Figure S1A-C and [12]) are down-regulated over the course of development in all four species, with the exception of *AqARGOS*, which is barely expressed (not shown), and *AqAIL6*, which has fairly consistent expression in DS1-DS4 but begins to decrease between DS4 and DS5 (Fig. 9a). Several of these genes have slightly lower expression in *A. ecalcarata*, including one of two *AquilegiaANT* homologs, *AqANT.1*, an *AIL* gene, *AqAIL5*, and *AqJAG*.

Negative regulators of cell proliferation in *A. thaliana* petals include *BIG BROTHER* (*BB*), *DA1*, the *TCP* genes *TCP4* and *TCP5*, and the cyclin-dependent kinase (CDK) inhibitor genes *KIP RELATED PROTEIN 4* (*KRP4*) and *KRP2* (reviewed in [6]). Although it may be expected that these genes would show the opposite pattern of expression from the cell-proliferation promoters, only a few of the *Aquilegia* homologs (see Additional file 1, Figure S1D-F and [29]) showed a pattern of increasing expression approaching DS5 (Fig. 9b). Two *KRP*-like genes, *AqKRP2*-like and *AqKRP*-like showed a strong increase in expression, especially between DS4 and DS5. Both of theses genes were also expressed more highly in *A. ecalcarata* than the spurred taxa. A third *KRP*-like gene with homology to *KRP4* actually sharply decreased in expression during later developmental stages in all taxa assessed. Aside from the two *KRP*-like genes previously mentioned, *AqDA1* is the only other candidate cell division repressor that appears to be increasing in expression toward DS5. The homolog of *Arabidopsis TCP4*, *AqTCP4*, was expressed at fairly consistent levels across all stages in the spurred taxa, but showed a substantial increase in expression in *A. ecalcarata* at DS3 and DS4, while *AqTCP5* showed a gradual decrease in expression across all taxa, but a slightly higher level of expression in *A. ecalcarata*. *Aquilegia* has two *BB* homologs, one that is expressed at a low level in all taxa and shows a pattern of decreasing expression over development (*AqBB.1*), and one that has fairly steady expression in the spurred taxa across phase I, but starts with a higher level of expression in *A. ecalcarata*(*AqBB.2*; Fig. 9b).

## Discussion

### Several components of spur morphological variation are established during phase I of petal development

Variation in the shape of plant organs is determined by combinatorial differences in cell number and cell shape. The *Aquilegia* petal has evolved substantial morphological variation, particularly in several aspects of nectar spur shape, including length, width, and curvature. Our current understanding of the development of the *Aquilegia* nectar spur involves two phases, an early mitotic phase (Phase I) and a later cell expansion phase (Phase II; [19, 29]). Despite differences in adult morphology, detailed ontogenetic studies using SEM have shown that from petal initiation through the earliest stages of Phase I, petals of a spurred species, *A. olympica*, and those of the spurless species, *A. ecalcarata*, are quite similar in shape [26]. In other species comparisons, differences in spur length at maturity have been largely attributed to differential cell elongation during phase II of development [19], however, the developmental basis of other aspects of spur shape have not been studied in detail. Based on the sampling conducted here, discernible differences in several axes of shape, particularly width and curvature, are apparent quite early in Phase I of petal development, suggesting that cell division plays a role in determining their differences. By examining gene expression across early petal development in several species with variable morphologies, this study allows us to identify gene expression modules that appear to be conserved across petals with diverse morphologies as well as modules that differ in correlation with variation in spur morphology. Further, this comparative approach may provide insight into the developmental processes that underlie the morphologies.

### Patterns of gene expression across development and between spurred taxa and *A. ecalcarata* suggest a heterochronic shift in petal development between spurred taxa and *A. ecalcarata*

**Fig. 10:**
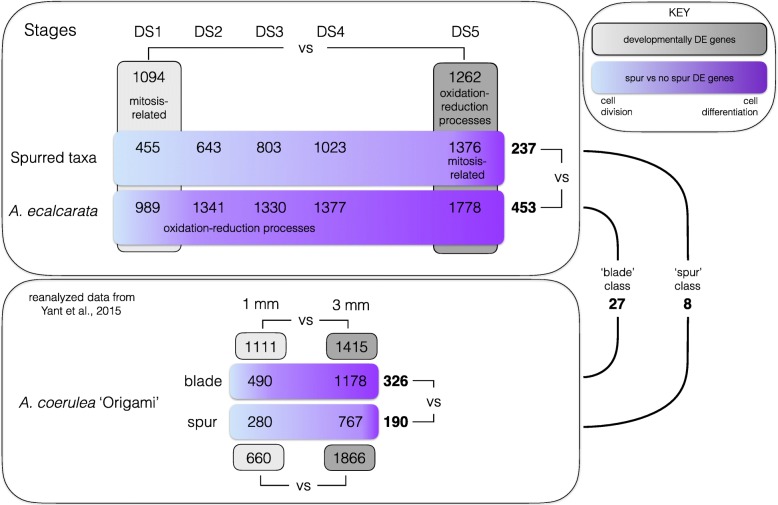
Summary of gene expression patterns. The upper panel summarizes results from the current data set and the lower panel summarizes results from Yant et al., 2015. Grey boxes show genes DE between developmental stages. Purple boxes show genes DE between samples with and without spur tissue. Upper panel: Between DS1 and DS5, more genes are commonly up-regulated late in development across all taxa (*n*=1262) than down-regulated (*n*=1094). Genes expressed early in development are enriched for GO terms related to mitosis while genes expressed later in development are enriched for GO terms related to oxidation-reduction processes (grey boxes). The number of genes DE between the three spurred taxa and *A. ecalcarata* increases across developmental stages (purple boxes). Late in development (DS5), genes related to mitosis are over-represented in the spurred taxa, while early in development (DS1-DS4), genes related to oxidation-reduction processes are over-represented in *A. ecalcarata*. More genes are commonly up-regulated in *A. ecalcarata* relative to the spurred taxa at all developmental stages (*n*=453 vs. *n*=237). Bottom panel: In *A. coerulea* ‘Origami’, between the 1 mm and 3 mm stages, more genes are up-regulated than down-regulated through development in both blade (*n*=1415 vs *n*=1111) and spur (*n*=1866 vs *n*=660) tissue (grey boxes). Between blade and spur tissue, a greater number of genes is up-regulated in blade tissue relative to spur tissue at both the 1 mm (*n*=490 vs *n*=280) and 3 mm stages (*n*=1178 vs *n*=767), and commonly across both stages (*n*=326 vs *n*=190; purple boxes). Identifying loci commonly DE across both panels, only 27 genes are commonly up-regulated in ‘blade’ class tissue and only 8 genes are commonly up-regulated in ‘spur’ class tissue

Several patterns emerge when we consider all of the expression data analyzed (summarized in Fig. 10). Over the course of petal development, GO enrichment analysis of genes commonly DE across all four taxa detected a pattern of declining expression of genes involved in mitotic activity. This pattern was also supported by correlations between WGCNA modules and developmental stages. Modules with eigengenes highly positively correlated with DS1 (and negatively correlated with DS5; MDs 3, 4, and 11) are enriched for GO terms related to mitosis. This finding of a decrease in mitotic activity throughout development is consistent both with petal developmental patterns in other model systems and with previous studies in *Aquilegia* [6, 19, 29]. In contrast to this pattern, an enrichment of genes involved in oxidation-reduction processes were found to be up-regulated at later developmental stages in all four taxa. This is supported by both the DE analyses between DS1 and DS5 and by the WGCNA analyses where several modules with eigengenes highly positively correlated with DS5 (and negatively correlated with DS1, MDs 21 and 23) are enriched for loci involved in oxidation-reduction processes.

Exploring the types of genes that are differentially expressed between the spurred taxa and *A. ecalcarata* detected up-regulation of loci with GO categories related to mitosis in the spurred taxa, but only at DS5 (summarized in Fig. 10). As *Aquilegia* petals develop, they begin to transition from cell division to expansion and differentiation, starting at the distal margin of the blade [19, 29]. In spurred taxa, this transition progresses from the petal margins toward the nascent nectary, with cell division persisting longest in the spur itself. Considering the pattern seen in the developmental comparisons and what is known about cellular processes during spur development, the increased expression of genes related to mitosis in the spurred taxa relative to *A. ecalcarata* suggests that the entire *A. ecalcarata* petal shifts into the differentiation phase at an earlier time point than in spurred taxa. Another pattern that supports this assertion is that there is an enrichment of loci involved with oxidation-reduction processes expressed more highly late in development (DS5) when considering all taxa, while when considering the loci DE between the spurred taxa and *A. ecalcarata* across development, there is an enrichment for loci with these processes at early stages (DS1-DS4) in *A. ecalcarata* (Fig. 10). A closer examination of genes with oxidation-reduction GO categorization in the developmental and spurred/spurlesss comparisons revealed a number of cytochrome P450 monooxygenases (CYPs) that at a molecular level function through heme/iron binding and oxidation [24, 28]. Although CYPs have similar molecular functions, they comprise the largest enzymatic gene family in plants and have evolved to play diverse roles in an array of cellular, developmental, and metabolic functions from hormone synthesis to pigment production [24, 28]. Many of these CYP functions appear to be important in differentiated cell types, rather than undifferentiated mitotically active cells. Thus, the up-regulation of CYPs earlier in *A. ecalcarata* development may be consistent with our hypothesis that these petals are accelerated in their differentiation relative to those in spurred species. Although the WGCNA identified a module that is highly correlated with the presence or absence of spurs (MD15), this module showed no GO enrichment.

A curious result that emerged when examining the genes that are commonly up-regulated across development in only spurred taxa was the enrichment of genes involved in photosynthetic processes. This was also seen in WGCNA module 20, which contains genes expressed more highly in spurred taxa at DS5 and is enriched for GO terms related to photosynthesis. While this enrichment of photosynthetic genes seems perplexing given that petals are generally not considered photosynthetic organs, we hypothesize that this result is likely an indirect consequence of spur development, rather than a cause. During phase I of *A. ecalcarata* development, the entire petal is shaded from light by the enclosing sepals. In the spurred taxa, however, elongation of the petal spurs causes them to emerge from the bud and become exposed to direct light, likely inducing baseline expression of photosynthetic loci (Additional file 1, Figure S4). Thus, this appears to be a background temporal component of spur development rather than a controlling factor.

### A small number of genes are consistently differentially expressed between petal tissue with spurs and without spurs

Although one might predict that more genes would be required for the development of the nectar spur, given its more complex three-dimensional structure compared to laminar petals and blades, a number of lines of evidence indicate that the number of loci required for early spur development may actually be relatively small. Considering the PCA conducted across the set of genes DE between DS1 and DS5 in any taxon (Fig. 3b), one might have expected that the presence or absence of spurs would be a major PC resulting in the grouping of samples from the spurred taxa apart from *A. ecalcarata*. However, none of the first 10 principle components examined capture such variation. The first principal component clustered samples by developmental stage, which is not surprising given how genes were selected for inclusion in the analysis. The second PC in this data set groups samples by geographic origin, with the Eurasian taxa clustering together and the North American taxa clustering together. This suggests that phylogenetic relatedness explains more shared developmental differences in gene expression in our data set than whether or not a nectar spur is produced. Several scenarios may contribute to this phenomenon. This pattern may indicate that relatively few genes are necessary to make a nectar spur and, thus, loci that consistently vary between the spurred taxa and *A. ecalcarata* do not explain a significant proportion of variation in this data set. Not mutually exclusive to this possibility, it may be that the genes important for spur production do not have variable expression levels across phase I of development and therefore were not captured in this set of analyses. Regardless, this result underscores the importance of sampling more than two species across divergent lineages for this type of study since, for instance, a pairwise comparison of *A. ecalcarata* and any single spurred species might have primarily identified loci that differ due to phylogenetic divergence rather than their morphological differences.

Given that crucial loci in nectar spur development may not be differentially expressed between DS1 and DS5, we also compared expression differences between the spurred taxa and *A. ecalcarata* at each of our developmental stages. These comparisons showed that during the earliest stages of spur development (DS1-DS3), there are *fewer* genes up-regulated in the spurred species than in *A. ecalcarata*. One possible explanation may be related to the proportion of differentiating cells in the petals of *A. ecalcarata* versus the spurred species. Given that the *A. ecalcarata* petal does not produce a spur and is entirely composed of blade tissue, a greater proportion of the *A. ecalcarata* petal may be in the differentiation phase relative to an equivalent sized petal that is producing a spur. Although a large set of genes are necessary for mitosis, the differentiation of cells into many specialized types may require the up-regulation of an even greater number of loci. In support of this hypothesis, several comparisons from the *A. coerulea* ‘Origami’ expression data set show a similar pattern. Comparing expression between the blade and the spur showed more loci are up-regulated in the blade (Fig. 10 and Additional file 2, Table S12) and developmental comparisons between the 1mm and 3mm blade and spur cup tissue samples demonstrated that a greater number of genes are up-regulated in both tissues at the later developmental stage (Fig. 10 and Additional file 2, Table S11). Another consistent data point is seen in the expression of *AqTCP4* (Fig. 9). Previous studies have found that *AqTCP4* is critical for the cell division to cell expansion/differentiation transition in *Aquilegia* and is expressed in a wave that starts at the blade margin and progresses towards the spur tip [29]. However, in the comparison between the four sampled taxa, *AqTCP4* is observed to peak during DS3-4 in *A. ecalcarata* while it is present at lower levels in the three spurred taxa. This is likely due to the absence of the prolonged proliferation needed for spur development, but may also reflect a developmental acceleration in *A. ecalcarata* relative to the spurred taxa, representing a heterochronic shift. Therefore, we infer that the greater up-regulation of genes in *A. ecalcarata* petals suggests that, across the organ, a larger proportion of cells have blade identity and have transitioned to expansion and differentiation. This pattern is likely to change over development as a greater proportion of the spurred petal begins to differentiate, including into cell types that are not present in the blade, such as trichomes and cells associated with the complex nectary [7].

Along these lines, it was surprising to find that *STY* homologs were not strongly differentially expressed between *A. ecalcarata* and the spurred taxa, given the absence of nectaries in *A. ecalcarata*. This may reflect the fact that *in situ* expression studies have revealed early expression of *STY* homologs at the distal tip of developing petals [13], which is consistent with the more deeply conserved role for *STY* homologs in controlling auxin homeostasis in lateral organs. It is likely that this distal expression domain is also present in *A. ecalcarata* during the early stages sampled here, making the detection of differential expression in whole petals more difficult than in the previous study where dissected blade and spur tissues were compared at later stages. Even so, there is a discernible trend of increasing *AqSTY* expression across time in the spurred species relative to *A. ecalcarata*.

While a relatively small number of genes were found to be consistently differentially expressed in entire early petals between the three spurred species and *A. ecalcarata*, an even smaller number of these genes were also found to be differentially expressed between the blade and the spur tissue of *A. coerulea* ‘Origami’. Only 35 genes showed consistent differential expression in what we term the ‘blade’ and ‘spur’ comparison classes between these studies, with 27 genes falling into the ‘blade’ class and 8 genes in the ‘spur’ class. Key loci in the development of nectar spurs are likely to act by prolonging mitosis in the spur cup, but none of these 35 loci are homologs of genes known to regulate the transition from cell division to differentiation in *Arabidopsis* by promoting or repressing mitosis. This list of 35 loci also does not contain several genes that are known to be necessary for proper spur formation. For example, the *Aquilegia* homolog of *JAG* has been shown to promote cell proliferation in petals as well as other organs [12]. In the current dataset, *AqJAG* is expressed at somewhat lower levels in *A. ecalcarata* versus the spurred taxa, but the temporal expression dynamics across the five stages are quite similar (Fig. 9a). Of course while not all of these 35 genes may have a direct role in spur development, we expect that genes with such a role are among this short list. Although it may be surprising that so few genes appear to consistently demarcate the ‘blade’ and ‘spur’, genetic crosses conducted in the 1960s between *A. ecalcarata* and multiple spurred taxa suggest that spur-loss is achieved in *A. ecalcarata* by altering only one or two loci [17, 18] and that this is done without grossly affecting other aspects of petal morphology. Thus, while a greater number of genes may be necessary for differentiation of spurs late in development, a small number of genes appear to be crucial for the localization and extension of the mitotic process early in spur development.

## Conclusions

The evolution of a novel trait that allows a lineage to radiate across a new adaptive landscape profoundly affects patterns of diversification. The *Aquilegia* nectar spur represents such a key innovation and understanding the genetic and developmental basis of this evolutionary novelty will provide insight into this important evolutionary process. While prior work has largely focused on late stage developmental processes involved in nectar spur variation, this study took a comparative gene expression approach across four species and a horticultural variety to identify core genetic constituents involved in the early stages of nectar spur development. These analyses indicate that prolonging mitosis is a key process in the early development of the *Aquilegia* nectar spur and identified a list of only 35 candidate loci that are consistently associated with the presence or absence of nectar spur tissue in *Aquilegia* petals. This list of 35 loci provides a manageable set of genes on which to focus future research efforts aimed at understanding how this novel trait evolved.

## Methods

### Tissue collection

Four species of *Aquilegia* - *A. ecalcarata*, *A. sibirica*, *A. formosa*, and *A. chrysantha* - were acquired as flowering plants (*n*=10 per species) from Suncrest Nurseries (Watsonville, CA). After acquisition, plants were kept in the same greenhouse bay under partially shaded natural light conditions and cooling fans turning on at 25^∘^C during the month of April at the University of California, Santa Barbara greenhouse facility. Petals from individual floral buds ranging between floral developmental stages 8 and 11 (see [1]) were dissected from the bud, imaged, and flash frozen with 4-5 petals collected per bud. Samples were randomly collected across species, plant, and developmental stage between 11 am and 3 pm daily across an ∼ 2-week time span in order to randomize circadian effects. After petal collection, images of all of the petal samples were used to identify and divide the samples into 5 developmental sub-stages (DS1-DS5) based on bud and petal morphology (Fig. 2) and three samples collected from different plants were selected for each species and developmental stage to represent biological replicates.

### RNA isolation and sequencing library preparation

RNA was isolated from three petal samples per species and developmental stage using the Qiagen RNeasy Micro kit (Qiagen, Valencia, CA, USA) for the earliest four stages and the Ambion PureLink RNA Mini kit for the latest stage (Life Technologies, Grand Island, NY, USA). RNA quantification was assessed using the Qubit 2.0 Fluorometer (Life Technologies, Grand Island, NY, USA) and quality was assessed using the 2200 TapeStation Instrument (Agilent Technologies, Santa Clara, CA, USA) at the UC Santa Barbara Biological Nanostructures Laboratory. Sequencing libraries were constructed using half reactions of the KAPA Stranded mRNA-Seq kit (KAPA Biosystems, Wilmington, MA, USA) with Illumina TruSeq indexing adaptors (Illumina Inc., San Diego, CA, USA). Libraries were quantified using qPCR, and pooled into sets of 20 samples each (each set had one sample of each species at each developmental stage), aiming for equal representation across samples. Each set was sequenced on a lane on the Illumina HiSeq4000 as 50-bp single end reads at the UC Davis Genome Center. Several samples that did not generate adequate sequence coverage initially were repooled and sequenced again with an additional lane of sequencing. Raw read data was deposited to the Sequence Read Archive (SRA) with sample accessions and read counts provided in Additional file 2, Table S1.

### Sequence processing and differential expression analyses

Three biological replicates per species and developmental stage were sequenced. Sequence data were aligned to the *Aquilegia coerulea* ‘Goldsmith’ v3.1 reference transcriptome (https://phytozome.jgi.doe.gov/) and processed to generate read counts per transcript as described in Filiault et al. (2). The R package edgeR was used to normalize read counts and to conduct tests for differential gene expression using pairwise comparisons (either between developmental stages of the same species or between species at the same developmental stage) using three replicate samples for each species and developmental stage [21]. For each comparison, read counts for each sample were normalized using the trimmed mean of M-values method (TMM, via the calcNormFactors command in edgeR) and transcripts with counts per million (cpm) greater than one in at least three samples were used to estimate dispersions and test for differential expression between sets of three biological replicates using the exact test [22, 23]. Transcripts with a false discovery rate (FDR) less than 0.05 were considered significantly DE.

### Gene ontology (GO) enrichment

Tests for GO enrichment were conducted using the R package goseq which considers gene length bias when determining significance of GO over-representation [16, 30]. Bias in the probability of identifying a gene as DE based on gene length was assessed using the probability weighting function (pwf) and the Wallenius approximation was used to determine over- and under-represented GO categories. The *p*-values were adjusted for multiple tests using the Benjamini and Hochberg (BH) method. When testing for GO enrichment in DE gene sets, DE gene sets were either compared to the set of genes identified as expressed at any developmental time point in any species (gene sets DE in DS1 vs DS5 and WGCNA modules, see below) or the set of genes identified as expressed in any species at a particular developmental stage (gene sets DE between *A. ecalcarata* and the spurred taxa at particular developmental stages). A gene was considered expressed if it had a cpm greater than one in three or more samples. Transcript GO annotations came from the *A. coerulea* “Goldsmith” v3.1 reference genome annotation (https://phytozome.jgi.doe.gov/).

### Principal component analysis (PCA)

Two sets of principal component analyses were conducted across all samples, one including any transcript identified as DE between DS1 and DS5 in any species, and one using transcripts commonly DE between all of the spurred taxa and *A. ecalcarata* at any developmental time point. For each analysis, z-scores were generated for each transcript across the distribution in all samples (*n*=60) using the R scale function [20]. As different samples had different total read outputs from sequencing, we first normalized the reads across each sample before calculating z-scores using the calcNormFactors function of edgeR [21]. Transcript z-scores were used to conduct PCA in R using the prcomp function [20]. Z-scores ranged from -4.11 to 7.62 for the developmentally DE genes and from -4.48 to 7.62 for the spurred versus *A. ecalcarata* comparison. Transcripts in which a sample had a value greater than 4.5 standard deviations from the mean were considered outliers and were filtered prior to conducting PCA.

### Weighted gene correlation network analysis (WGCNA)

Weighted gene correlation network analysis was conducted using the R package WGCNA following the methodology laid out in the WGCNA tutorials [9, 10] on the set of genes considered expressed in our data set (genes with a cpm greater than one in at least three of the 60 samples - four species, five developmental time points, three replicates). The soft thresholding power used to calculate adjacency was chosen as the lowest power for which the scale-free topology fit index reached 0.9 (*β* = 12). Adjacency was transformed into the Topological Overlap Matrix and dissimilarity was calculated. Hierarchical clustering was conducted on dissimilarities. Branch cutting was initially performed using Dynamic Tree Cut with a minimum module size of 20. The first principal component of the standardized expression profiles of loci in a module (the module eigengene) was calculated and initial modules with highly co-expressed genes (eigengenes with co-expression correlations greater than 0.75) were then merged to generate final cluster modules. Correlations between modules (the eigengene) and traits (i.e., developmental stage, species, geography) were calculated to identify significant associations.

### Identification of *Aquilegia* candidate genes based on homology to *A. thaliana* genes

For several candidate genes (*JAG, TCP4*), prior homology assessments have been made (*AqTCP4* - [29]; *AqJAG* - [12]). In order to identify *Aquilegia* homologs of additional *A. thaliana* candidate genes, Phytozome (https://phytozome.jgi.doe.gov/) was used to BLAST the protein of the *A. thaliana* candidate against a protein database including the following species: *Amborella trichopoda* (v1.0), *Medicago truncatula* (Mt4.0v1), *Vitis vinifera* (Genoscope.12x), *Arabidopsis thaliana* (TAIR10), *Oryza sativa* (v7_JGI), Zea mays (ENSEMBL-18), *Mimulus guttatus* (v.2.0), *Solanum lycopersicum* (iTAG2.4), and *Aquilegia coerulea* “Goldsmith” (v3.1). Protein sequences were aligned using ClustalW with the BLOSUM cost matrix in Geneious (version 9.1.6, https://www.geneious.com) and Neighbor Joining trees using the Jukes-Cantor genetic distance model were constructed using the Geneious Tree Builder (provided in Additional file 1, Figure S1 A-H). Minimal alterations were made to the protein alignment, but large, uninformative insertions were removed before analyses.

## Additional files

Additional file 1 supplemental figures. **Figure S1.** Gene trees of protein alignments to determine homology between *Arabidopsis* petal development genes and *Aquilegia* genes. A. Gene tree of genes related to *MONOPTEROS/AUXIN RESPONSE FACTOR 5*. B. Gene tree of genes related to *AINTEGUMENTA* and *AINTEGUMENTA-LIKE*. C. Gene tree of genes related to *GROWTH REGULATING FACTOR*. D. Gene tree of genes related to *BIG BROTHER*. E. Gene tree of genes related to *DA1*. F. Gene tree of genes related to *KIP RELATED PROTEINS*. **Figure S2.** Gene expression plots of *Aquilegia STYLISH* homologs *AqSTY1*, *AqSTY2*, and *AqLRP* across development. *A. ecalcarata* = lavendar; *A. sibirica* = blue; *A. formosa* = red, *A. chrysantha* = yellow. **Figure S3.** Gene expression plots of the loci up-regulated in the ‘blade’ class across development. *A. ecalcarata* = lavendar; *A. sibirica* = blue; *A. formosa* = red, *A. chrysantha* = yellow. **Figure S4.** Image of a floral bud at DS5 showing how the nectar spur is exposed to light at later developmental stages. (PDF 4494 kb)

Additional file 2 supplemental tables. **Table S1.** Summary of samples, SRA accession, and read counts for each biological replicate of each species at each developmental stage. **Table S2.** Summary of the number of genes up- or down-regulated within a species between consecutive developmental stages. **Table S3.** GO enrichment of genes commonly DE between DS1 and DS5 in the spurred taxa only. Ontology category abbreviations - BP : biological process, MF : molecular function, CC : cellular component. A GO category was deemed enriched relative to all annotated genes in the *Aquilegia* reference transcriptome if the *p*-value adjusted for multiple comparisons using the Benjamini-Hochberg (BH) method was less than 0.05. **Table S4.** Gene counts commonly up or down regulated in one species relative to the other three at each developmental stage. **Table S5.** GO enrichment of WGCNA modules. Ontology category abbreviations - BP : biological process, MF : molecular function, CC : cellular component. A GO category was deemed enriched relative to all annotated genes in the *Aquilegia* reference transcriptome if the *p*-value adjusted for multiple comparisons using the Benjamini-Hochberg (BH) method was less than 0.05. **Table S6.** Number of overlapping developmentally DE genes (DS1 vs DS5) with modules identified by WGCNA. **Table S7.** Number of overlapping genes DE between spurred taxa and *A. ecalcarata* with WGCNA modules. **Table S8.** Number of overlapping genes uniquely DE in *A. sibirica* with WGCNA modules. **Table S9.** Number of overlapping genes uniquely DE in *A. formosa* with WGCNA modules. **Table S10.** Number of overlapping genes uniquely DE in *A. chrysantha* with WGCNA modules. **Table S11.** Number of genes DE between the 1mm and 3 mm stage in blade and spur cup tissue of *A. coerulea* ‘Origami’ in reanalysis of Yant et al., 2015 data. **Table S12.** Number of genes DE between blade and spur cup tissue at 1 mm and 3 mm stages in reanalysis of Yant et al., 2015 data. **Table S13.** Overlap of DE genes between the blade and spur cup at 1 mm and 3 mm in reanalysis of Yant et al., 2015 data with genes DE between *A. ecalcarata* and the spurred taxa at stages DS3 and DS4 in the current study, respectively. (PDF 159 kb)

Additional file 3 genes DE between DS1 and DS5 in all taxa. File format: Tab delimited text file. (TXT 46 kb)

Additional file 4 genes DE between DS1 and DS5 only in spurred taxa. File format: Tab delimited text file. (TXT 17 kb)

Additional file 5 genes used in developmental PCA. File format: Tab delimited text file. (TXT 176 kb)

Additional file 6 genes used in spurred taxa vs *A. ecalcarata* PCA. File format: Tab delimited text file. (TXT 79 kb)

Additional file 7 genes commonly DE between spurred taxa and *A. ecalcarata* at all developmental stages. File format: Tab delimited text file. (TXT 21 kb)

Additional file 8 genes in each WGCNA module. File format: Tab delimited text file. (TXT 438 kb)

Additional file 9 gene DE between blade and spur tissue at 1 mm stage. File format: Tab delimited text file. (TXT 18 kb)

Additional file 10 gene DE between blade and spur tissue at 3 mm stage. File format: Tab delimited text file. (TXT 45 kb)

Additional file 11 genes commonly DE in ‘blade’ and ‘spur’ classes, including protein categorization information. File format: Tab delimited text file. (TXT 29 kb)

## Data Availability

Raw sequence data is available from the National Center for Biotechnology Information’s (NCBI) Sequence Read Archive (SRA) under the submission PRJNA515749.

## Abbreviations

*AIL*: *AINTEGUMENTA-LIKE*
*ANT*: *AINTEGUMENTA*
*Aq*: *Aquilegia*
*ARF*: *AUXIN RESPONSE FACTOR*
*ARGOS*: *AUXIN-RELATED GENE INVOLVED IN ORGAN GROWTH*
*At*: *Arabidopsis thaliana*
*BB*: *BIG BROTHER*
BH: Benjamini and Hochberg
CDK: Cyclin-dependent kinase
CIB: Cryptochrome 2-interacting basic helix-loop-helix
cpm: Counts per million
CYP: Cytochrome P450
DE: Differentially expressed
DS: Developmental stage
*GIF*: *GRF-INTERACTING FACTOR*
GO: Gene ontology
HSF: Heat shock factor
*JAG*: *JAGGED*
KNOX: Knotted 1-like homeobox, *KRP*: *KIP RELATED PROTEIN*
*LRP*: *LATERAL ROOT PRIMORDIUM*
LRR: Leucine rich repeat
MD: Module
*MP*: *MONOPTEROS*
OVP: OVATE family protein
PC: Principle component
PCA: Principle component analysis
Pfam: Protein family
RNAseq: RNA sequencing
SRA: Short read archive
*STY*: *STYLISH*
*TCP*: *TEOSINTE BRANCHED/CYCLOIDEA/PCF*
TMM: Trimmed mean of M-values
WGCNA: Weighted gene correlation network analysis
