# Supplementary material for: Comparative transcriptomics of early petal development across four diverse species of Aquilegia reveal few genes consistently associated with nectar spur development
Source: BMC Genomics. 2019 Aug 22;20:668. doi: 10.1186/s12864-019-6002-9 (PMC6704642; doi:10.1186/s12864-019-6002-9)
Supplement: Supplementary file 1 — supplemental figures. Figure S1. Gene trees of protein alignments to determine homology between Arabidopsis petal development genes and Aquilegia genes. A. Gene tree of genes related to MONOPTEROS/AUXIN RESPONSE FACTOR 5. B. Gene tree of genes related to AINTEGUMENTA and AINTEGUMENTA-LIKE. C. Gene tree of genes related to GROWTH REGULATING FACTOR. D. Gene tree of genes related to BIG BROTHER. E. Gene tree of genes related to DA1. F. Gene tree of genes related to KIP RELATED PROTEINS. Figure S2. Gene expression plots of Aquilegia STYLISH homologs AqSTY1, AqSTY2, and AqLRP across development. A. ecalcarata = lavendar; A. sibirica = blue; A. formosa = red, A. chrysantha = yellow. Figure S3. Gene expression plots of the loci up-regulated in the ‘blade’ class across development. A. ecalcarata = lavendar; A. sibirica = blue; A. formosa = red, A. chrysantha = yellow. Figure S4. Image of a floral bud at DS5 showing how the nectar spur is exposed to light at later developmental stages. (PDF 4494 kb) [file 12864_2019_6002_MOESM1_ESM.pdf]

Supplemental Figure 1A

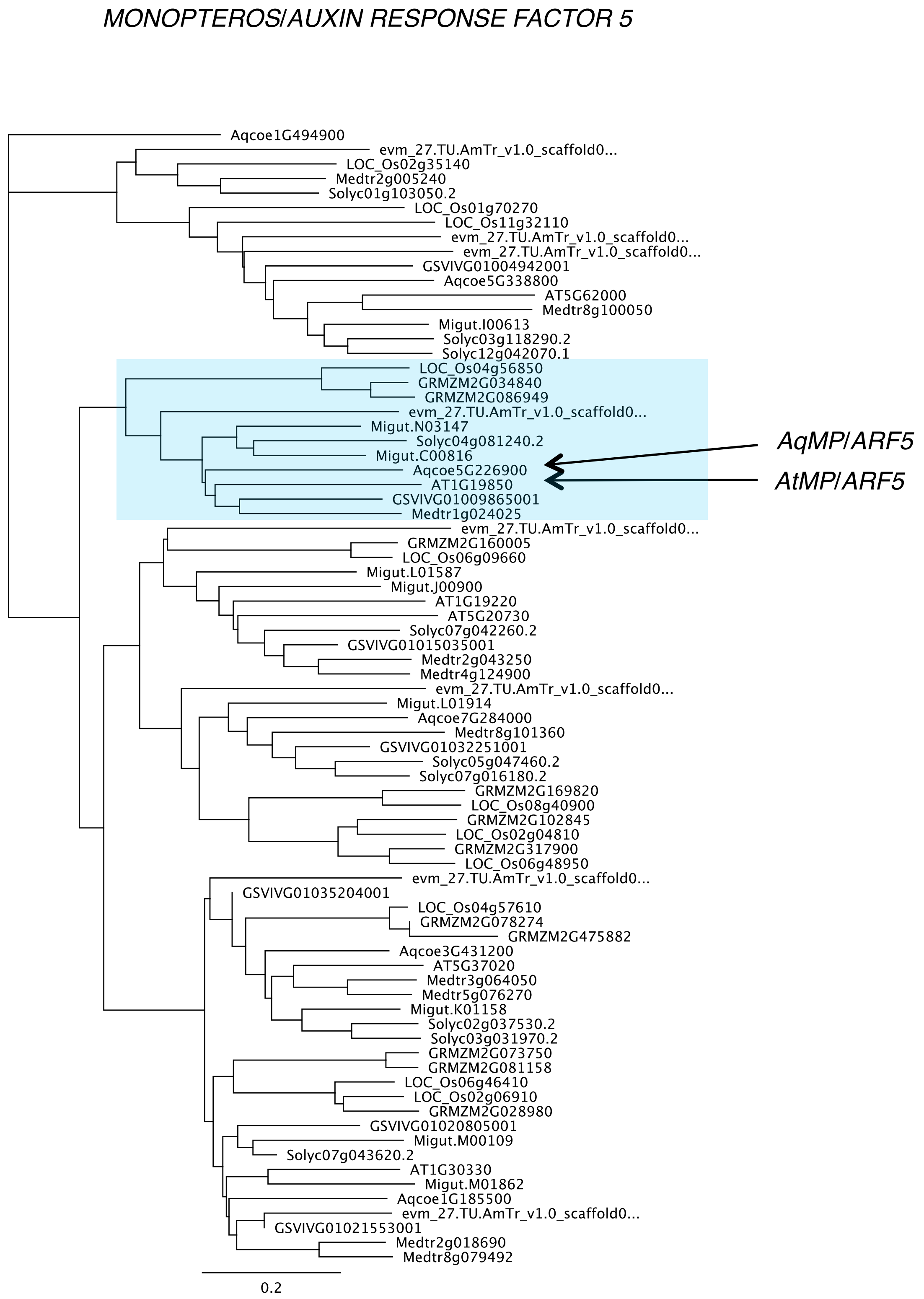

Supplemental Figure 1B

AINTEGUMENTA and *AINTEGUMENTA-LIKE* GENES

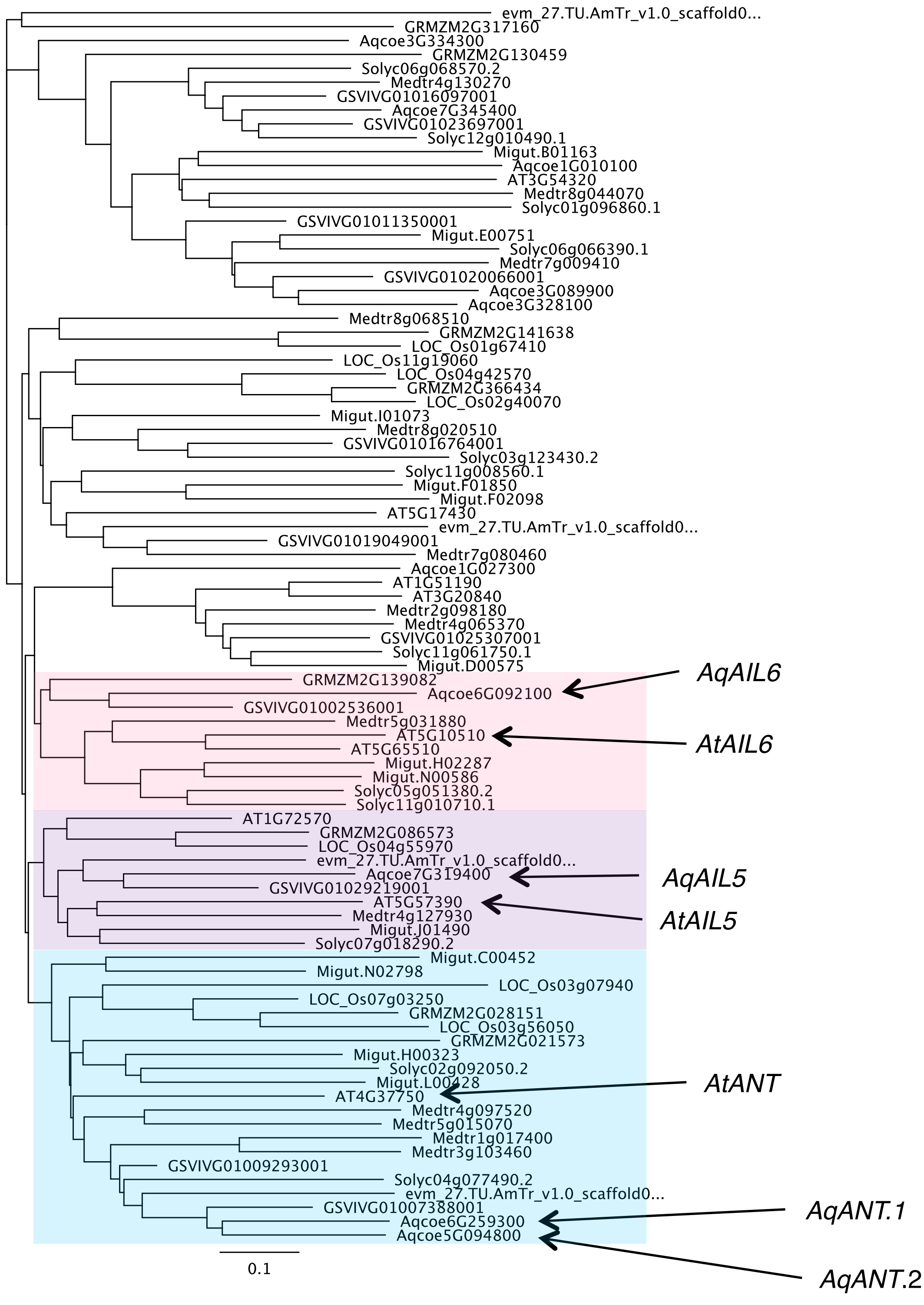

Supplemental Figure 1C

GROWTH REGULATING FACTOR GENES

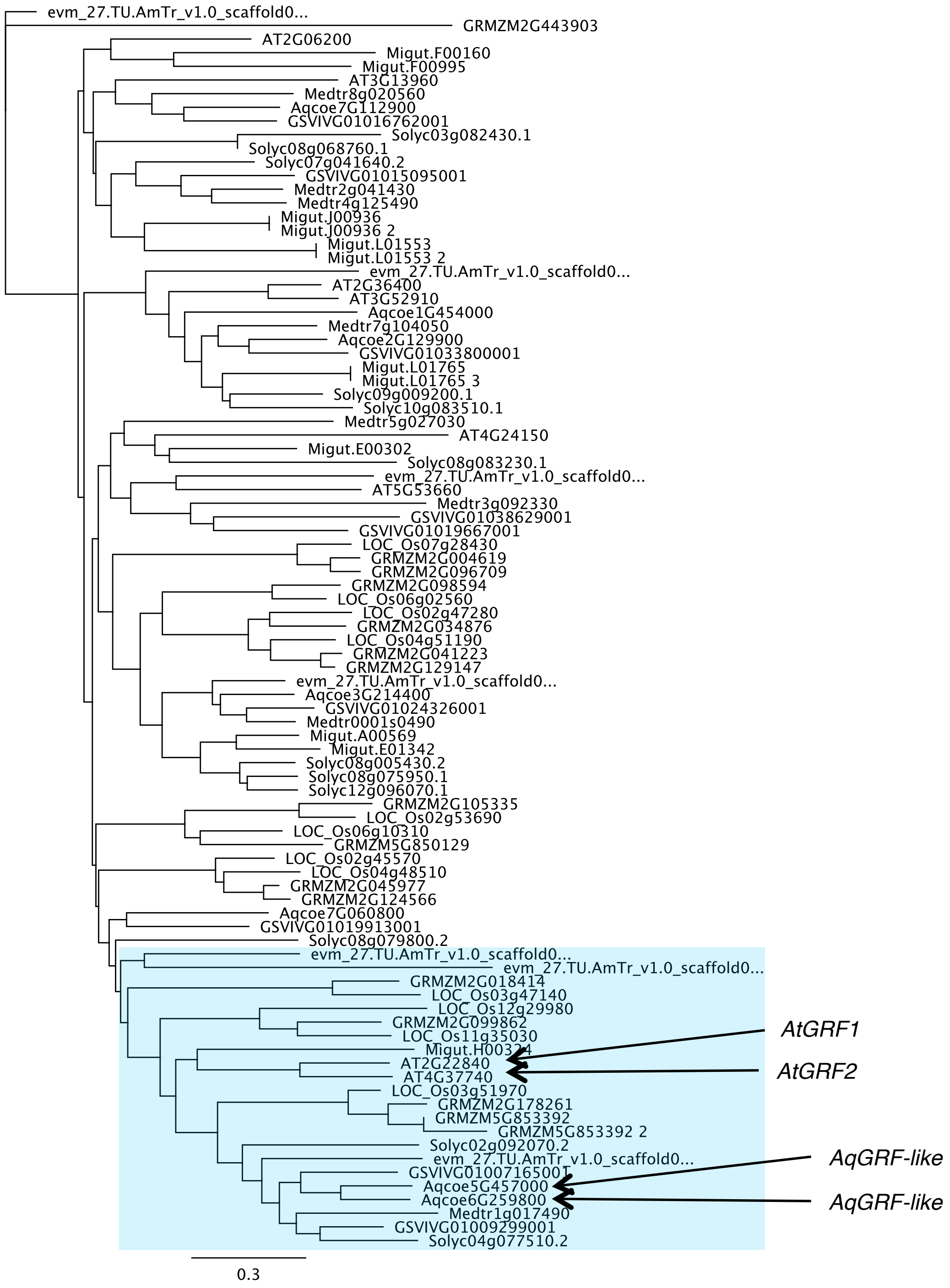

Supplemental Figure 1D

BIG BROTHER GENES

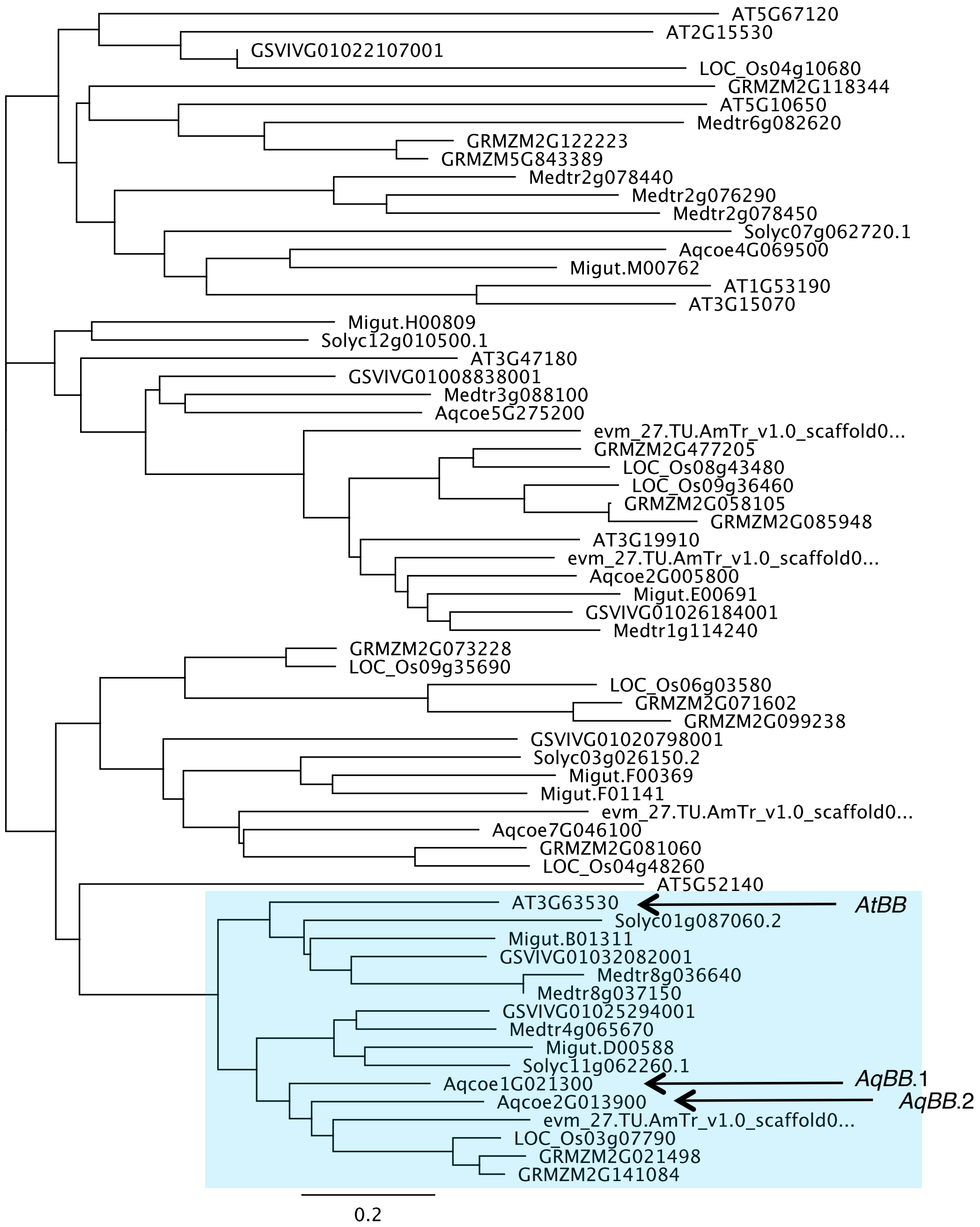

Supplemental Figure 1E

DA1 GENES

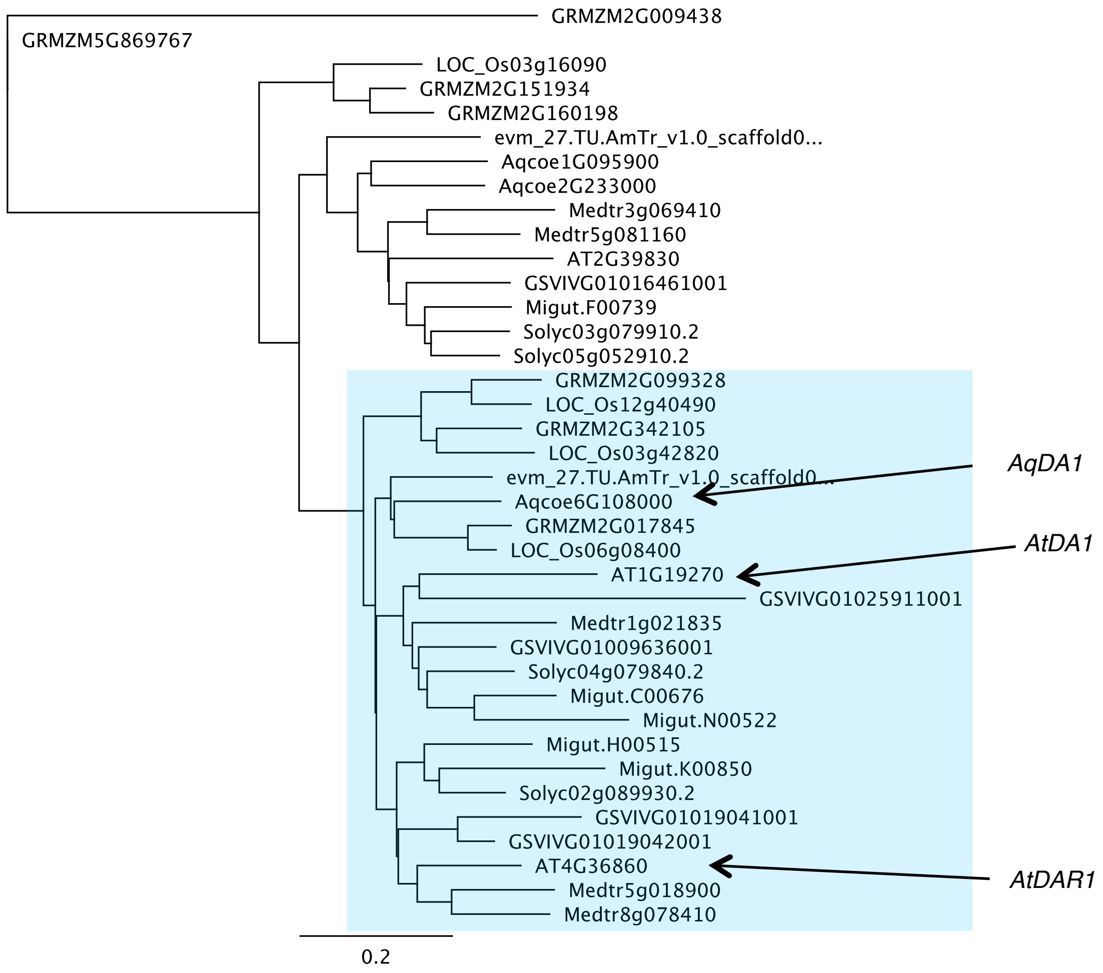

Supplemental Figure 1F

KIP RELATED PROTEIN GENES

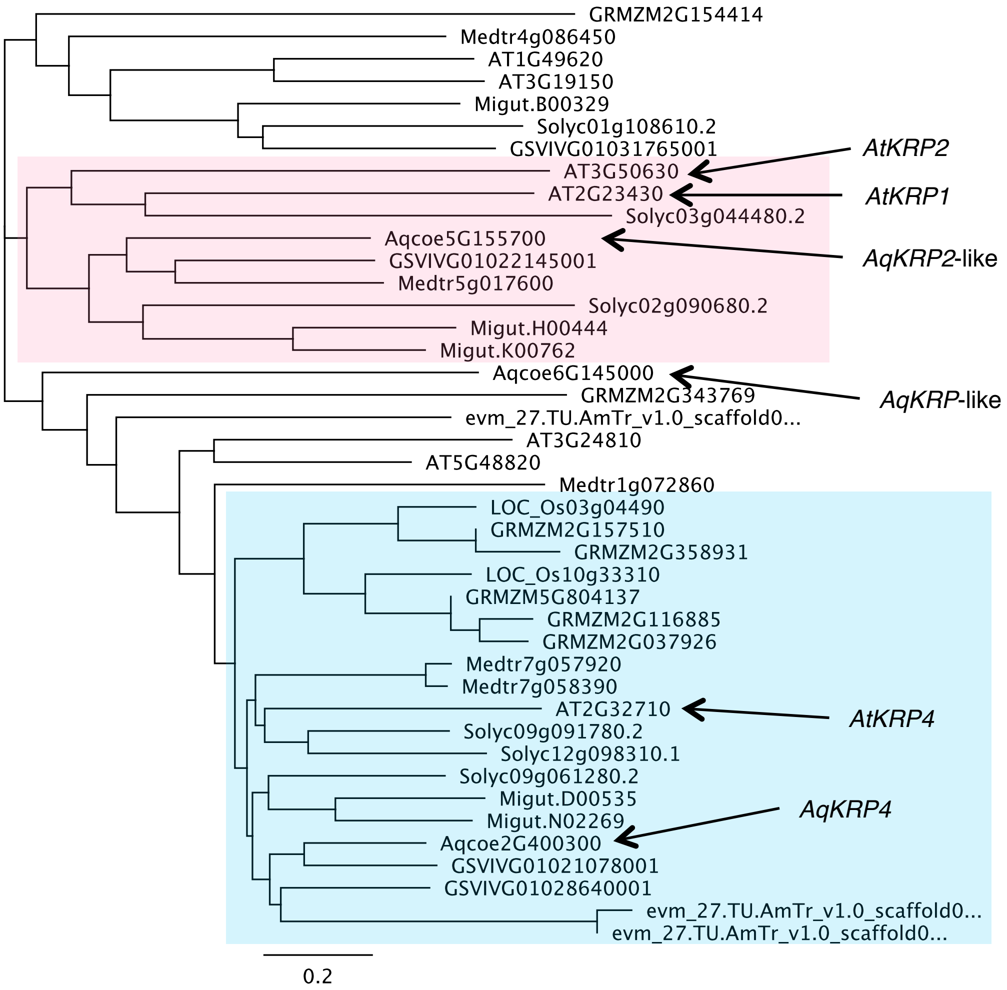

Supplemental Figure 2

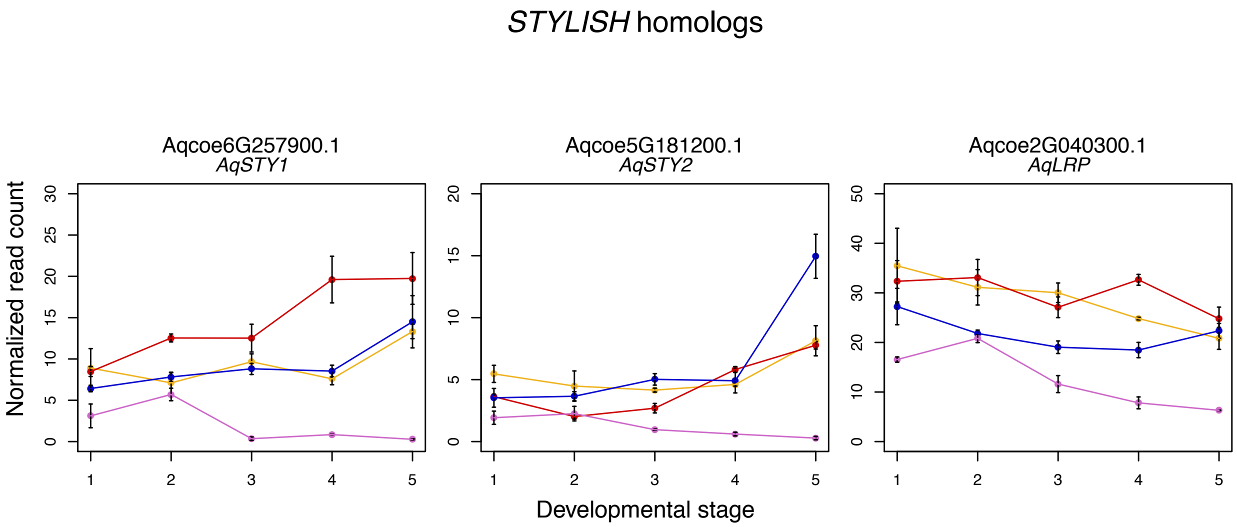

Supplemental Figure 3

Genes up−regulated in blade and *A. ecalcarata*

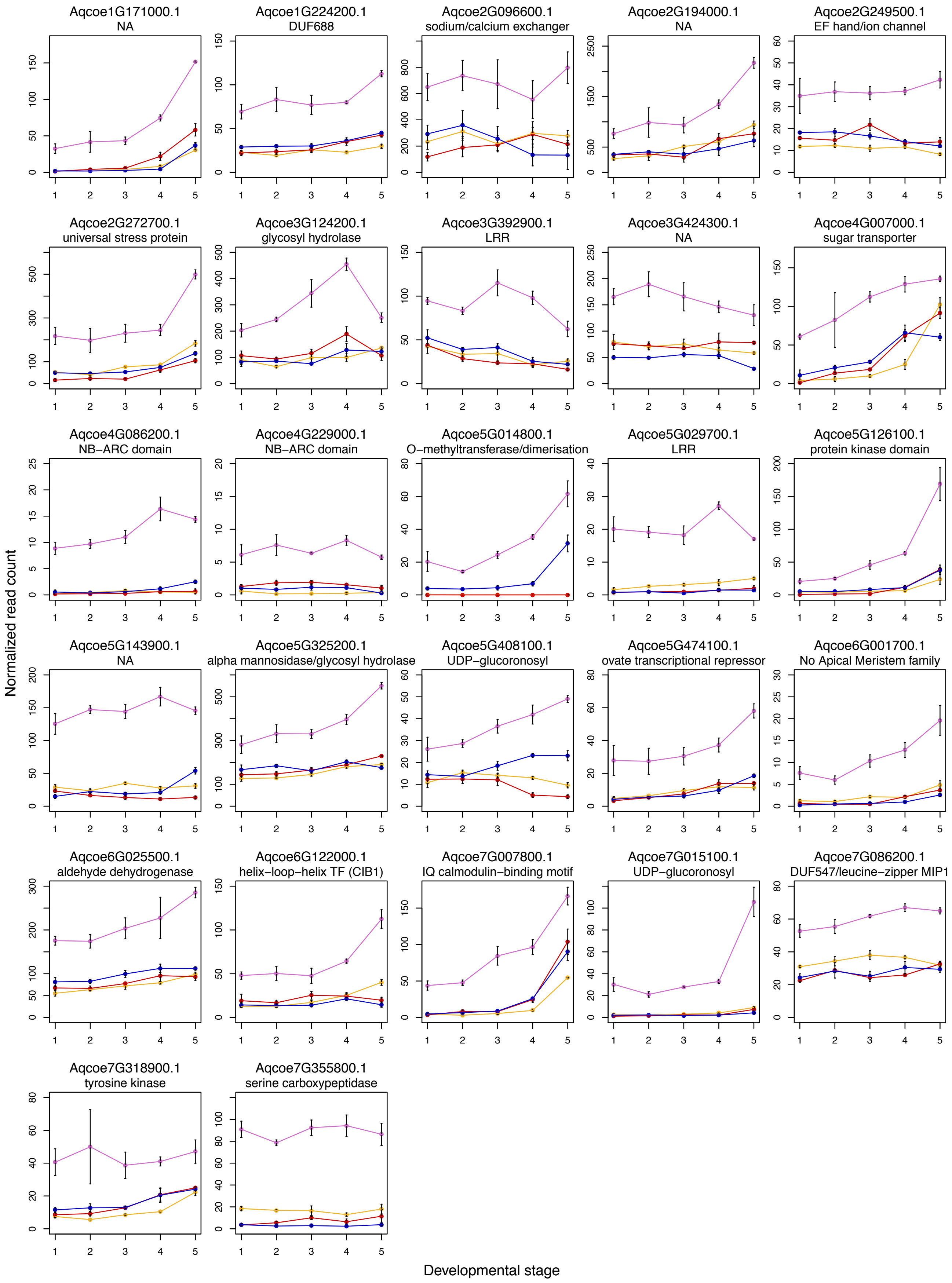

Supplemental Figure 4

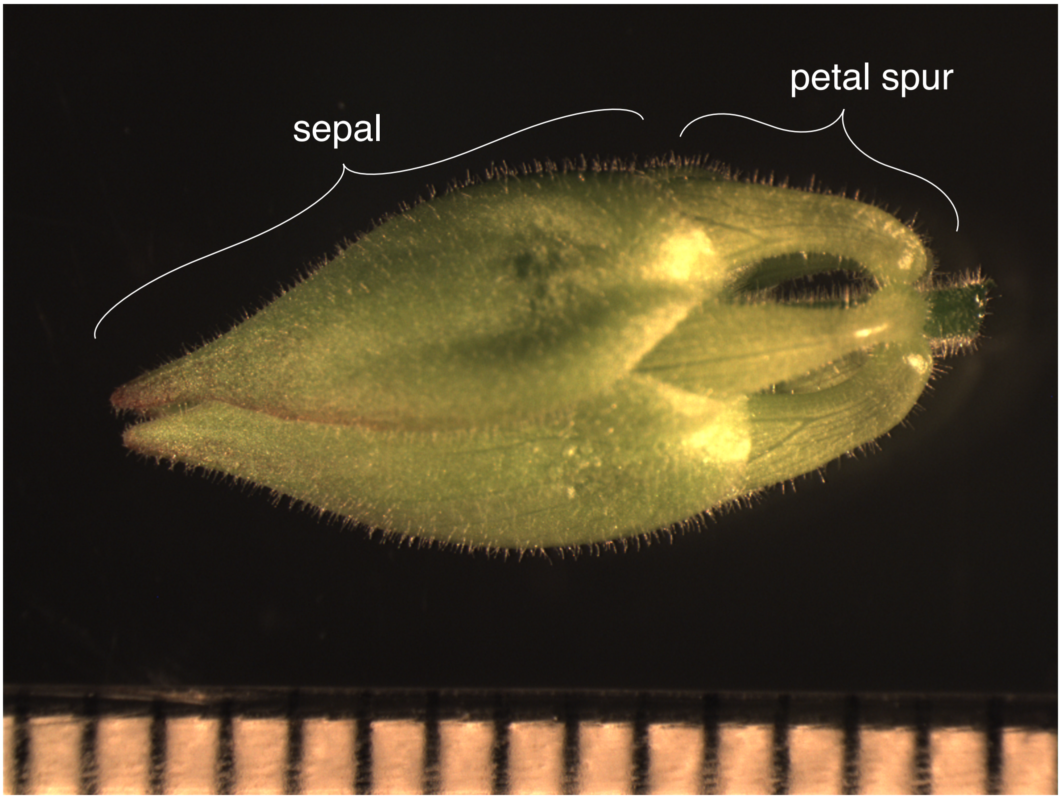

Image demonstrating how the nectar spur is exposed to light at later developmental stages
