# Supplementary material for: Comparative transcriptomics of early petal development across four diverse species of Aquilegia reveal few genes consistently associated with nectar spur development
Source: BMC Genomics. 2019 Aug 22;20:668. doi: 10.1186/s12864-019-6002-9 (PMC6704642; doi:10.1186/s12864-019-6002-9)
Supplement: Supplementary file 2 — supplemental tables. Table S1. Summary of samples, SRA accession, and read counts for each biological replicate of each species at each developmental stage. Table S2. Summary of the number of genes up- or down-regulated within a species between consecutive developmental stages. Table S3. GO enrichment of genes commonly DE between DS1 and DS5 in the spurred taxa only. Ontology category abbreviations - BP : biological process, MF : molecular function, CC : cellular component. A GO category was deemed enriched relative to all annotated genes in the Aquilegia reference transcriptome if the p-value adjusted for multiple comparisons using the Benjamini-Hochberg (BH) method was less than 0.05. Table S4. Gene counts commonly up or down regulated in one species relative to the other three at each developmental stage. Table S5. GO enrichment of WGCNA modules. Ontology category abbreviations - BP : biological process, MF : molecular function, CC : cellular component. A GO category was deemed enriched relative to all annotated genes in the Aquilegia reference transcriptome if the p-value adjusted for multiple comparisons using the Benjamini-Hochberg (BH) method was less than 0.05. Table S6. Number of overlapping developmentally DE genes (DS1 vs DS5) with modules identified by WGCNA. Table S7. Number of overlapping genes DE between spurred taxa and A. ecalcarata with WGCNA modules. Table S8. Number of overlapping genes uniquely DE in A. sibirica with WGCNA modules. Table S9. Number of overlapping genes uniquely DE in A. formosa with WGCNA modules. Table S10. Number of overlapping genes uniquely DE in A. chrysantha with WGCNA modules. Table S11. Number of genes DE between the 1mm and 3 mm stage in blade and spur cup tissue of A. coerulea ‘Origami’ in reanalysis of Yant et al., 2015 data. Table S12. Number of genes DE between blade and spur cup tissue at 1 mm and 3 mm stages in reanalysis of Yant et al., 2015 data. Table S13. Overlap of DE genes between the blade and spur cup at 1 [file 12864_2019_6002_MOESM2_ESM.pdf]

Supp. Table 1. Sample Accession and Read Counts

Species	Developmental Stage	Sample Name	SRA Accession	Reads
A. ecalcarata	**1**	E1.1	SAMN10767326	8598859
		E1.2	SAMN10767327	13695171
		E11.05	SAMN10767328	27731596
	**2**	E2.2	SAMN10767329	21904948
		E2.4	SAMN10767330	18484940
		E2.1	SAMN10767331	13954378
	**3**	E3.2	SAMN10767332	9867680
		E3.1	SAMN10767333	13914482
		E3.4	SAMN10767334	15249358
	**4**	E4.1	SAMN10767335	11318457
		E4.4	SAMN10767336	19966385
		E4.2	SAMN10767337	27802795
	**5**	E5.1	SAMN10767338	18968148
		E5.2	SAMN10767339	27307407
		E5.4	SAMN10767340	19275310
A. sibirica	**1**	S1.1	SAMN10767341	15835638
		S1.3	SAMN10767342	19520477
		S1.4	SAMN10767343	17638008
	**2**	S2.1	SAMN10767344	29073489
		S2.2	SAMN10767345	13787797
		S2.3	SAMN10767346	27276138
	**3**	S3.1	SAMN10767347	28243206
		S3.2	SAMN10767348	16190719
		S3.3	SAMN10767349	13507606
	**4**	S4.2	SAMN10767350	24070070
		S4.3	SAMN10767351	26132376
		S4.4	SAMN10767352	11672852
	**5**	S5.1	SAMN10767353	23336879
		S5.2	SAMN10767354	73675476
		S5.3	SAMN10767355	16278285
A. formosa	**1**	F1.2	SAMN10767356	28166793
		F1.3	SAMN10767357	14745020
		F1.4	SAMN10767358	17853569
	**2**	F2.1	SAMN10767359	22510106
		F2.3	SAMN10767360	14564974
		F2.4	SAMN10767361	32735055
	**3**	F3.4	SAMN10767362	18213810
		F6.02	SAMN10767363	29008498
		F6.03	SAMN10767364	28437542
	**4**	F4.2	SAMN10767365	26629630
		F4.3	SAMN10767366	22336223

Species	Developmental Stage	Sample Name	SRA Accession	Reads
		F4.4	SAMN10767367	11466805
	5	F5.1	SAMN10767368	11779719
		F5.2	SAMN10767369	11895596
		F5.3	SAMN10767370	15659147
A. chrysantha	1	C1.1	SAMN10767371	21689516
		C1.2	SAMN10767372	14249331
		C1.4	SAMN10767373	27928554
	2	C2.1	SAMN10767374	25960239
		C2.3	SAMN10767375	14099220
		C2.4	SAMN10767376	15568431
	3	C3.2	SAMN10767377	32961220
		C3.3	SAMN10767378	16849173
		C3.4	SAMN10767379	16503316
	4	C4.1	SAMN10767380	11277825
		C4.3	SAMN10767381	18337656
		C4.4	SAMN10767382	13846669
	5	C5.2	SAMN10767383	25341020
		C5.3	SAMN10767384	30708975
		C5.4	SAMN10767385	11143963

Supp. Table 2. Genes DE between developmental stages

	DS1 to DS2		DS2 to DS3		DS3 to DS4		DS4 to DS5	
Species	up	down	up	down	up	down	up	down
A. ecalcarata	14	3	87	107	48	42	3406	3115
A. sibirica	0	0	45	12	181	110	2443	1524
A. formosa	83	103	123	61	1673	790	459	190
A. chrysantha	13	2	87	36	28	11	2356	1201

Supp. Table 3. GO enrichment of genes DE through dev, spurred taxa only

Expression through development	GO category	term	ontology	BH adj p-value
up	GO:0015979	photosynthesis	BP	5.98E-19
	GO:0009765	photosynthesis, light harvesting	BP	2.84E-15
	GO:0009523	photosystem II	CC	9.26E-12
	GO:0009654	photosystem II oxygen evolving complex	CC	8.19E-09
	GO:0009522	photosystem I	CC	9.51E-07
	GO:0019898	extrinsic component of membrane	CC	2.03E-06
	GO:0009538	photosystem I reaction center	CC	3.84E-06
	GO:0016020	membrane	CC	6.66E-05
	GO:0016491	oxidoreductase activity	MF	0.00022
	GO:0005509	calcium ion binding	MF	0.00095
	GO:0055114	oxidation-reduction process	BP	0.00489
	GO:0019684	photosynthesis, light reaction	BP	0.006569
	GO:0042651	thylakoid membrane	CC	0.009459
	GO:0050661	NADP binding	MF	0.022969
	GO:0018580	nitronate monooxygenase activity	MF	0.031449
down	GO:0003735	structural constituent of ribosome	MF	8.27E-11
	GO:0006412	translation	BP	9.06E-11
	GO:0005840	ribosome	CC	9.58E-11
	GO:0005622	intracellular	CC	3.57E-09
	GO:0003676	nucleic acid binding	MF	6.32E-05

Supp. Table 4. Genes commonly up/down regulated in one species relative to the other three

	A. ecalcarata		***A. sibirica***		***A. formosa***		***A . chrysantha***	
**Dev**	**up**	**down**	**up**	**down**	**up**	**down**	**up**	**down**
**1**	989	455	675	470	414	254	621	319
**2**	1341	643	1129	723	662	385	786	587
**3**	1330	803	908	615	814	473	886	577
**4**	1377	1023	1175	753	925	381	1181	924
**5**	1778	1376	969	686	603	283	1087	728
**intersect**	453	237	346	209	169	58	288	149

Supp. Table 5. Module GO Enrichment

Module	GO category	term	ontology	BH adj p-value
MD1	GO:0004601	peroxidase activity	MF	0.03812
MD3	GO:0006412	translation	BP	6.35E-52
	GO:0003735	structural constituent of ribosome	MF	1.39E-48
	GO:0005840	ribosome	CC	1.81E-46
	GO:0005622	intracellular	CC	1.18E-25
	GO:0015934	large ribosomal subunit	CC	0.00011
	GO:0008033	tRNA processing	BP	0.01166
	GO:0003676	nucleic acid binding	MF	0.02947
	GO:0042254	ribosome biogenesis	BP	0.03824
	GO:0003723	RNA binding	MF	0.04363
MD4	GO:0003676	nucleic acid binding	MF	1.59E-13
	GO:0006260	DNA replication	BP	3.87E-12
	GO:0003677	DNA binding	MF	2.36E-11
	GO:0005634	nucleus	CC	1.36E-08
	GO:0008017	microtubule binding	MF	1.17E-06
	GO:0005871	kinesin complex	CC	3.72E-06
	GO:0003777	microtubule motor activity	MF	6.43E-06
	GO:0007018	microtubule-based movement	BP	6.43E-06
	GO:0003735	structural constituent of ribosome	MF	0.00436
	GO:0005622	intracellular	CC	0.00685
	GO:0005840	ribosome	CC	0.00685
	GO:0006412	translation	BP	0.00767
	GO:0006364	rRNA processing	BP	0.014357
	GO:0016592	mediator complex	CC	0.03782
MD6	GO:0000723	telomere maintenance	BP	0.00048
	GO:0003678	DNA helicase activity	MF	0.00056
	GO:0006281	DNA repair	BP	0.00318
MD7	GO:0004857	enzyme inhibitor activity	MF	1.40E-07
	GO:0005618	cell wall	CC	1.40E-06
	GO:0030599	pectinesterase activity	MF	1.40E-06
	GO:0042545	cell wall modification	BP	1.40E-06
	GO:0030570	pectate lyase activity	MF	0.01549
MD8	GO:0006281	DNA repair	BP	0.00095
	GO:0000723	telomere maintenance	BP	0.01078
	GO:0003678	DNA helicase activity	MF	0.01427
MD10	GO:0019684	photosynthesis, light reaction	BP	8.545E-09
	GO:0009539	photosystem II reaction center	CC	3.00E-06
	GO:0015979	photosynthesis	BP	1.11E-05
	GO:0009579	thylakoid	CC	0.00017
	GO:0009772	photosynthetic electron transport in photosystem II	BP	0.00017
	GO:0045156	electron transporter, transferring electrons within the cyclic electron transport pathway of photosynthesis activity	MF	0.00017
	[illegible]	[illegible]	[illegible]	[illegible]

Module	GO category	term	ontology	BH adj p-value
	GO:0009507	chloroplast	CC	0.00017
	GO:0017004	cytochrome complex assembly	BP	0.00017
	GO:0009521	photosystem	CC	0.00169
	GO:0009767	photosynthetic electron transport chain	BP	0.00169
	GO:0016168	chlorophyll binding	MF	0.00169
	GO:0015232	heme transporter activity	MF	0.00298
	GO:0015886	heme transport	BP	0.00298
	GO:0009523	photosystem II	CC	0.00736
	GO:0008137	NADH dehydrogenase (ubiquinone) activity	MF	0.01402
	GO:0009522	photosystem I	CC	0.01543
MD11	GO:0006412	translation	BP	1.67E-13
	GO:0003735	structural constituent of ribosome	MF	5.28E-13
	GO:0005840	ribosome	CC	5.28E-13
	GO:0005622	intracellular	CC	1.19E-09
	GO:0004298	threonine-type endopeptidase activity	MF	1.45E-06
	GO:0005839	proteasome core complex	CC	1.45E-06
	GO:0051603	proteolysis involved in cellular protein catabolic	BP	1.45E-06
	GO:0000786	process	CC	1.73E-06
	GO:0004175	nucleosome	MF	0.00259
	GO:0019773	endopeptidase activity	CC	0.00259
	GO:0006511	proteasome core complex, alpha-subunit complex	BP	0.00367
		ubiquitin-dependent protein catabolic process		
MD18	GO:0018580	nitronate monooxygenase activity	MF	0.00400
	GO:0008171	O-methyltransferase activity	MF	0.02604
MD19	GO:0004097	catechol oxidase activity	MF	0.00468
MD20	GO:0015979	photosynthesis	BP	3.23E-21
	GO:0009765	photosynthesis, light harvesting	BP	2.64E-10
	GO:0009523	photosystem II	CC	5.61E-10
	GO:0009654	photosystem II oxygen evolving complex	CC	1.25E-08
	GO:0019898	extrinsic component of membrane	CC	1.19E-07
	GO:0009522	photosystem I	CC	3.10E-07
	GO:0009538	photosystem I reaction center	CC	1.59E-06
	GO:0003735	structural constituent of ribosome	MF	1.14E-05
	GO:0006412	translation	BP	1.77E-05
	GO:0005840	ribosome	CC	1.77E-05
	GO:0005509	calcium ion binding	MF	0.00146
	GO:0006457	protein folding	BP	0.00257
	GO:0042651	thylakoid membrane	CC	0.00620
	GO:0015995	chlorophyll biosynthetic process	BP	0.01786
	GO:0016851	magnesium chelatase activity	MF	0.03022
MD21	GO:0055114	oxidation-reduction process	BP	1.52E-07
	GO:0016491	oxidoreductase activity	MF	9.83E-06
	GO:0016747	transferase activity, transferring acyl groups other than amino-acyl groups	MF	0.00500
	GO:0016758	transferase activity, transferring hexosyl groups	MF	0.00857
	GO:0008152	metabolic process	BP	0.01933
	GO:0006633	fatty acid biosynthetic process	BP	0.02692

Module	GO category	term	ontology	BH adj p-value
MD22	GO:0009733<br>GO:0005975	response to auxin<br>carbohydrate metabolic process	BP<br>BP	0.00796<br>0.02778
MD23	GO:0055114<br>GO:0016705<br><br>GO:0030117<br>GO:0006886<br>GO:0005506<br>GO:0005198<br>GO:0020037	oxidation-reduction process<br>oxidoreductase activity, acting on paired donors, with incorporation or reduction of molecular oxygen<br>membrane coat<br>intracellular protein transport<br>iron ion binding<br>structural molecule activity<br>heme binding	BP<br>MF<br><br>CC<br>BP<br>MF<br>MF<br>MF	0.00925<br>0.00925<br><br>0.01680<br>0.01873<br>0.02296<br>0.02296<br>0.02296
MD25	GO:0003700	transcription factor activity, sequence-specific DNA binding	MF	0.00049

Supp. Table 6. Developmentally DE genes by module

Module	Genes per module	Up-regulated from DS1 to DS5			Down-regulated from DS1 to DS5		
		DE genes	% of DE	% of module	DE genes	% of DE	% of module
MD1	124	3	< 1	2.4	0	0	0
MD2	66	0	0	0	0	0	0
MD3	1027	19	1.5	1.9	**185**	**17**	**18**
MD4	2619	98	7.8	3.7	**547**	**50**	**21**
MD5	855	1	< 1	< 1	2	< 1	< 1
MD6	1296	6	< 1	< 1	5	< 1	< 1
MD7	166	0	0	0	0	0	0
MD8	1956	4	< 1	< 1	14	1.3	< 1
MD9	308	0	0	0	0	0	0
MD10	643	0	0	0	1	< 1	< 1
MD11	748	5	< 1	< 1	**174**	**16**	**23**
MD12	352	1	< 1	< 1	7	< 1	2
MD13	267	0	0	0	0	0	0
MD14	59	0	0	0	0	0	0
MD15	2984	22	1.7	< 1	16	1.5	< 1
MD16	154	6	< 1	3.9	0	0	0
MD17	57	9	< 1	16	0	0	0
MD18	101	11	< 1	11	0	0	0
MD19	50	0	0	0	0	0	0
MD20	274	11	< 1	4	0	0	0
MD21	825	**160**	**13**	**19**	2	< 1	< 1
MD22	2468	**626**	**50**	**25**	99	9	4
MD23	1241	**274**	**22**	**22**	33	3	2.7
MD24	46	0	0	0	0	0	0
MD25	64	0	0	0	0	0	0
MD26	2172	6	< 1	< 1	9	< 1	< 1
MD27	53	0	0	0	0	0	0
NA	815	0	0	0	0	0	0

Supp. Table 7. Spurred taxa vs A. ecalcarata DE genes by module

Module	Genes per module	Up-regulated in spurred taxa			Up-regulated in *A. ecalcarata*		
		DE genes	% of DE	% of module	DE genes	% of DE	% of module
MD1	124	0	0	0	0	0	0
MD2	66	0	0	0	0	0	0
MD3	1027	1	< 1	< 1	0	0	0
MD4	2619	28	12	1.1	5	1.1	< 1
MD5	855	9	3.8	1.1	0	0	0
MD6	1296	7	3	< 1	0	0	0
MD7	166	0	0	0	0	0	0
MD8	1956	24	10	1.2	0	0	0
MD9	308	7	3	2.3	2	< 1	< 1
MD10	643	0	0	0	3	< 1	< 1
MD11	748	0	0	0	0	0	0
MD12	352	2	< 1	< 1	0	0	0
MD13	267	0	0	0	5	1.1	1.9
MD14	59	0	0	0	2	< 1	3.4
**MD15**	**2984**	**135**	**57**	**4.5**	**404**	**89**	**14**
MD16	154	0	0	0	0	0	0
MD17	57	1	< 1	1.8	0	0	0
MD18	101	0	0	0	0	0	0
MD19	50	0	0	0	0	0	0
MD20	274	0	0	0	0	0	0
MD21	825	3	1.3	< 1	0	0	0
MD22	2468	0	0	0	32	7.1	1.3
MD23	1241	4	1.7	< 1	0	0	0
MD24	46	1	< 1	2.2	0	0	0
MD25	64	0	0	0	0	0	0
MD26	2172	15	6.3	< 1	0	0	0
MD27	53	0	0	0	0	0	0
NA	815	0	0	0	0	0	0

Supp. Table 8. Genes DE uniquely in A. sibirica by module

Module	Genes per module	Unique DE in *A. sibirica*					
		up			down		
		n	% n	% of module	n	% of n	% of module
MD1	124	11	< 1	8.9	8	< 1	6.5
MD2	66	4	< 1	6.1	3	< 1	4.5
MD3	1027	235	8.8	23	99	4.6	9.6
MD4	2619	108	4	4.1	181	8.5	6.9
MD5	855	34	1.3	4	65	3	7.6
MD6	1296	31	1.2	2.4	91	4.3	7
MD7	166	7	< 1	4.2	1	< 1	0.6
MD8	1956	44	1.6	2.2	172	8.1	8.8
MD9	308	28	1	9.1	20	< 1	6.5
MD10	643	41	1.5	6.4	42	2	6.5
MD11	748	60	2.2	8	60	2.8	8
MD12	352	55	2.1	16	49	2.3	14
MD13	267	8	< 1	3	29	1.4	11
MD14	59	0	0	0	0	0	0
MD15	2984	124	4.6	4.2	269	13	9
MD16	154	5	< 1	3.2	16	< 1	10
MD17	57	11	< 1	19	0	0	0
MD18	101	0	0	0	18	< 1	18
MD19	50	4	< 1	8	5	< 1	10
MD20	274	6	< 1	2.2	40	1.9	15
MD21	825	24	< 1	2.9	111	5.2	13
MD22	2468	103	3.9	4.2	246	12	10
MD23	1241	433	**16**	35	107	5	8.6
MD24	46	28	1	61	3	< 1	6.5
MD25	64	32	1.2	50	0	0	0
**MD26**	**2172**	**1160**	**43**	**53**	**445**	**21**	**20**
MD27	53	33	1.2	62	4	< 1	7.5
NA	815	39	1.5	4.8	50	2.3	6.1

Supp. Table 9. Genes DE uniquely in A. formosa by module

Module	Genes per module	Unique DE in *A. formosa*					
		up			down		
		n	% n	% of module	n	% of n	% of module
MD1	124	11	< 1	8.9	6	< 1	4.8
MD2	66	4	< 1	6.1	3	< 1	4.5
MD3	1027	39	1.7	3.8	35	2.1	3.4
MD4	2619	352	15	13	164	10	6.3
MD5	855	149	6.5	17	69	4.2	8.1
**MD6**	**1296**	**720**	**31**	**56**	**178**	**11**	**14**
MD7	166	19	< 1	11	4	< 1	2.4
MD8	1956	30	1.3	1.5	73	4.5	3.7
MD9	308	22	< 1	7.1	16	< 1	5.2
MD10	643	17	< 1	2.6	113	6.9	18
MD11	748	36	1.6	4.8	84	5.1	11
MD12	352	33	1.4	9.4	36	2.2	10
MD13	267	6	< 1	2.2	69	4.2	26
MD14	59	2	< 1	3.4	19	1.2	32
MD15	2984	109	4.7	3.7	185	11	6.2
MD16	154	10	< 1	6.5	9	< 1	5.8
MD17	57	3	< 1	5.3	4	< 1	7
MD18	101	4	< 1	4	10	< 1	9.9
MD19	50	14	< 1	28	1	< 1	2
MD20	274	37	1.6	14	32	2	12
MD21	825	330	14	40	39	2.4	4.7
MD22	2468	198	8.6	8	210	13	8.5
MD23	1241	57	2.5	4.6	73	4.5	5.9
MD24	46	5	< 1	11	1	< 1	2.2
MD25	64	0	0	0	19	1.2	30
MD26	2172	56	2.4	2.6	148	9.1	6.8
MD27	53	1	< 1	1.9	0	0	0
NA	815	38	1.7	4.7	35	2.1	4.3

Supp. Table 10. Genes DE uniquely in A. chrysantha by module

Module	Genes per module	Unique DE in *A. chrysantha*					
		up			down		
		n	% of n	% of module	n	% of n	% of module
MD1	124	8	< 1	6.5	12	< 1	9.7
MD2	66	13	< 1	20	2	< 1	3
MD3	1027	71	2.3	6.9	81	3.2	7.9
MD4	2619	633	21	24	214	8.5	8.2
MD5	855	111	3.6	13	52	2.1	6.1
MD6	1296	29	< 1	2.2	93	3.7	7.2
MD7	166	31	1	19	8	< 1	4.8
**MD8**	**1956**	**1154**	**38**	**59**	**299**	**12**	**15**
MD9	308	31	1	10	37	1.5	12
MD10	643	27	< 1	4.2	82	3.3	13
MD11	748	71	2.3	9.5	57	2.3	7.6
MD12	352	29	< 1	8.2	25	1	7.1
MD13	267	67	2.2	25	27	1.1	10
MD14	59	3	< 1	5.1	4	< 1	6.8
MD15	2984	159	5.2	5.3	233	9.3	7.8
MD16	154	35	1.1	23	3	< 1	1.9
MD17	57	17	< 1	30	5	< 1	8.8
MD18	101	74	2.4	73	2	< 1	2
MD19	50	1	< 1	2	6	< 1	12
MD20	274	28	< 1	10	77	3.1	28
MD21	825	94	3.1	11	62	2.5	7.5
MD22	2468	170	5.5	6.9	540	21	22
MD23	1241	72	2.3	5.8	307	12	25
MD24	46	2	< 1	4.3	0	0	0
MD25	64	10	< 1	16	7	< 1	11
MD26	2172	84	2.7	3.9	206	8.2	9.5
MD27	53	2	< 1	3.8	5	< 1	9.4
NA	815	48	1.6	5.9	66	2.6	8.1

Sup. Table 11. Developmentally DE genes in blade and spur cup tissue

	1mm	VS	3mm
**blade**	1111		1415
**spur cup**	660		1866

Sup. Table 12. Tissue-specific DE genes at 1 mm and 3 mm stages

	blade	VS	spur cup
**1 mm**	490		280
**3 mm**	1178		767

Sup. Table 13. Overlap of blade vs. cup DE with A. ecalcarata vs. spurred

		blade		spur cup	
		A. ecalcarata	**spurred taxa**	***A. ecalcarata***	**spurred taxa**
**1 mm**		490		280	
	**intersect DS3**	102	21	21	43
**3 mm**		1178		767	
	**intersect DS4**	163	43	31	108
